# Identification and validation of tryptophan-related gene signatures to predict prognosis and immunotherapy response in lung adenocarcinoma reveals a critical role for PTTG1

**DOI:** 10.3389/fimmu.2024.1386427

**Published:** 2024-07-31

**Authors:** Ziqiang Wang, Jing Zhang, Chao Zuo, Huili Chen, Luyao Wang, Yiluo Xie, Hongyu Ma, Shengping Min, Xiaojing Wang, Chaoqun Lian

**Affiliations:** ^1^ Anhui Province Key Laboratory of Clinical and Preclinical Research in Respiratory Disease, Molecular Diagnosis Center, Joint Research Center for Regional Diseases of Institute of Health and Medicine (IHM), First Affiliated Hospital of Bengbu Medical University, Bengbu, China; ^2^ Research Center of Clinical Laboratory Science, Bengbu Medical University, Bengbu, China; ^3^ Department of Genetics, School of Life Sciences, Bengbu Medical University, Bengbu, China; ^4^ Department of Clinical Laboratory, Affiliated Hospital of Guilin Medical University, Guilin, China; ^5^ Department of Clinical Medicine, Bengbu Medical University, Bengbu, China

**Keywords:** lung adenocarcinoma, tryptophan metabolism, immunotherapy, prognostic model, tumor microenvironment

## Abstract

**Introduction:**

Tryptophan metabolism is strongly associated with immunosuppression and may influence lung adenocarcinoma prognosis as well as tumor microenvironment alterations.

**Methods:**

Sequencing datasets were obtained from The Cancer Genome Atlas (TCGA) and the Gene Expression Omnibus (GEO) database. Two different clusters were identified by consensus clustering, and prognostic models were established based on differentially expressed genes (DEGs) in the two clusters. We investigated differences in mutational landscapes, enrichment pathways, immune cell infiltration, and immunotherapy between high- and low-risk scoring groups. Single-cell sequencing data from Bischoff et al. were used to identify and quantify tryptophan metabolism, and model genes were comprehensively analyzed. Finally, PTTG1 was analyzed at the pan-cancer level by the pan-TCGA cohort.

**Results:**

Risk score was defined as an independent prognostic factor for lung adenocarcinoma and was effective in predicting immunotherapy response in patients with lung adenocarcinoma. PTTG1 is one of the key genes, and knockdown of PTTG1 *in vitro* decreases lung adenocarcinoma cell proliferation and migration and promotes apoptosis and down-regulation of tryptophan metabolism regulators in lung adenocarcinoma cells.

**Discussion:**

Our study revealed the pattern and molecular features of tryptophan metabolism in lung adenocarcinoma patients, established a model of tryptophan metabolism-associated lung adenocarcinoma prognosis, and explored the roles of PTTG1 in lung adenocarcinoma progression, EMT process, and tryptophan metabolism.

## Introduction

Lung cancer is the cancer with the highest incidence and mortality rate worldwide and remains the leading cause of cancer deaths (1, 2). Lung adenocarcinoma (LUAD) is the most common pathologic subtype of lung cancer, accounting for approximately 40% of all lung cancer cases (3). In most cases, tumors are found to be locally advanced or metastatic disease, and despite significant advances in combination treatment strategies for LUAD, the average 5-year survival rate for LUAD is approximately 15% (4). In recent years, the clinical use of immunotherapies targeting immune checkpoints has been shown to improve survival in advanced non-small cell lung cancer (NSCLC), but only some patients respond to them (5). Various biomarkers such as tumor mutational burden (TMB), PD-L1 expression, microsatellite instability (MSI), mismatch repair defects (dMMR), mutations in cancer driver genes, and immunogenetic signatures are widely used in clinical practice (6, 7). Novel markers such as circulating tumor cells (CTCs) and circulating tumor DNA (ctDNA) are also expected to be used to assess the efficacy of immune checkpoint inhibitors (ICIs) (8, 9). However, LUAD has molecular heterogeneity and diverse tumor microenvironment (TME) compositions, making it difficult to fully reflect the heterogeneous TME and thus predict immunotherapy efficacy (10, 11). Therefore, it is necessary to develop predictive models and identify new biomarkers to predict prognosis and treatment efficacy. Altered cellular metabolism is considered a hallmark of cancer, and previous studies have shown that glutamine and arginine metabolism are closely associated with macrophage activation and immunomodulation. And tumor-associated macrophages (TAMs), the most abundant immune component of TME, not only support tumor progression and metastasis but also cause further immunosuppression (12, 13). However, the molecular alterations and metabolic patterns of tryptophan in lung adenocarcinoma have not been fully investigated.

Tryptophan (TRP) is an essential amino acid. Tryptophan and its metabolites play key roles in a variety of physiological processes, ranging from cell growth and maintenance to coordinating the body’s response to the environment and diet (14). There is growing evidence that tryptophan catabolism is involved in immune tolerance and promotes responses to other antitumor agents through the Kynurenine (KYN) pathway (15). Indoleamine 2,3-dioxygenase (IDO) and tryptophan-2,3-dioxygenase (TDO) catalyze the same reaction and are rate-limiting enzymes in the kynurenine pathway (16). IDO has been shown to have immunosuppressive effects (17), and inhibition of IDO expression enhances antitumor immunity (18). Studies have shown that IDO has an immunosuppressive effect, and anti-tumor immunity can be enhanced by inhibiting the expression of IDO. Similar immunosuppressive effects of TDO have been demonstrated by inhibiting T cell proliferation and blocking immune cell infiltration (19, 20). IDO activity induces tryptophan depletion, leading to GCN2 activation and mTOR inhibition, which in turn leads to effector T cell incompetence. IDO expression is not only associated with a decrease in tumor-infiltrating lymphocytes (TILs) but also inhibits the antigen presentation response of T cells to dendritic cells (DCs) (21, 22). In patients with advanced NSCLC, higher IDO activity (kyn/trp ratio) predicts resistance to anti-PD-1 therapy (23). In addition, tryptophan degradation is thought to suppress immune cells through the formation of immunosuppressive tryptophan catabolic metabolites and tryptophan depletion (24). These studies suggest that tryptophan plays an important role in tumor progression and antitumor immune processes and that tryptophan metabolism has potential implications for immunotherapy of LUAD.

Pituitary tumor transforming gene 1 (PTTG1) is considered a proto-oncogene that promotes cell cycle progression, maintains chromosomal stability, and mediates *in vitro* transformation and *in vivo* tumorigenesis (7, 25). PTTG1 is highly expressed and associated with poor prognosis in a variety of cancers, including lung adenocarcinoma, hepatocellular carcinoma, breast cancer, and glioma, and its expression is positively correlated with tumor oncogenicity and significantly affects the ability of tumors to proliferate, migrate, and invade by upregulating cellular markers of epithelial-mesenchymal transition (EMT) and transcriptional factors that induce a malignant phenotype in tumors (26–29). In Zhou et al.’s study, PTTG1 was found to promote hepatocellular carcinoma cell proliferation and hepatocellular carcinoma progression by upregulating asparagine synthase (ASNS)-mediated asparagine metabolism and activating the mTOR pathway. While in LUAD, PTTG1 has been preliminarily shown to promote LUAD progression and inhibit LUAD growth and invasion by regulating TGFB1/SMAD3 signaling (30, 31). In LUAD, the association between PTTG1 and tryptophan metabolism and the potential mechanism during epithelial mesenchymal transition (EMT) have not been investigated.

In this study, we comprehensively analyzed the Cancer Genome Atlas (TCGA), the Gene Expression Omnibus (GEO) database, and the single-cell sequencing data from Bischoff et al. (32). in the LUAD dataset to explore the expression patterns and predictive potential of the tryptophan-related genes (TRPRGs). First, two distinct subtypes were identified by consistent clustering, with significant differences in metabolic alterations, biological processes, and immune characteristics between the subtypes. Next, we constructed TRP-associated prognostic features by Lasso Cox regression and validated them in multiple GEO cohorts. Then, we identified five independent immunotherapy cohorts to validate the predictive performance of immunotherapy efficacy. In addition, a comprehensive analysis of the five prognostic features was conducted, and finally, we identified a specific role in the tumor microenvironment based on the tryptophan metabolism-associated hub gene, PTTG1, and conducted a series of cellular experiments to validate the correlation between PTTG1 and tryptophan metabolism, as well as its promotional role in the process of proliferation, migration, EMT and apoptosis in LUAD.

## Methods

### Data collection and processing

Transcriptomic data, single nucleotide mutations (SNVs), and copy number mutations (CNVs) in LUAD patients were downloaded from The Cancer Genome Atlas (TCGA) (https://portal.gdc.cancer.gov/), selecting lung adenocarcinoma samples with complete survival information (n=500). In addition, we downloaded the GSE31210 (n=226), GSE50081 (n=127), and GSE30219 (n=278) datasets from the Gene Expression Omnibus (GEO) database (https://www.ncbi.nlm.nih.gov/geo/) as a validation cohort. To comprehensively investigate the metabolic pattern and predictive potential of tryptophan metabolism for LUAD, TRPRGs selected in this study were obtained from the MSigDB (https://www.gsea-msigdb.org/gsea/msigdb) database (33) including KEGG_TRYPTOPHAN_METABOLISM, REACTOME_TRYPTOPHAN_CATABOLISM and WP_TRY PTOPHAN_METABOLISM. After removing duplicate genes, a total of 51 TRPRGs were included in this study (**Supplementary Table 1**). Based on the patient’s overall survival (OS), we performed univariate Cox regression analysis using the “survival” R package to assess the prognostic value of the 51 TRPRGs in LUAD patients. Fourteen TRPRGs associated with OS were screened according to P<0.05 for subsequent analysis. Similarly, the pan-cancer analysis was based on RNA-seq data for 33 cancer types in the TCGA database and corresponding clinical information. The RNA-seq data type for processing TCGA was log2(TPM+1).

### Collection and processing of data for single-cell RNA-seq analysis

We used single-cell RNA sequencing data (50,093 transcriptomes after quality control and filtering) from 10 LUAD samples (5 normal lungs and 5 lung adenocarcinoma samples) from the single-cell sequencing data of Bischoff et al. Single-cell sequencing data were analyzed using the “Seurat” software package. Quality control (QC) was performed by retaining cells with less than 10% of mitochondrial genes and genes with expression ranging from 100 to 8000 in at least three cells. We then identified highly variable genes and set the number of highly variable genes to 2000 for subsequent analysis. The “Harmony” software package was used to remove batch effects. We constructed cell clusters using the “FindClusters” and “FindNeighbors” functions and visualized them using the “t-SNE” method. Finally, we performed cellular annotation based on the marker genes of different cell types.

The built-in function “AddModuleScore” in the Seurat package was used to quantify the activity of a specific set of genes in each cell. To analyze the differentially expressed genes (DEGs) between the two groups, we used the “FindMarkers” function in the Seurat package. The statistical significance of DEGs was calculated using the Wilcoxon test (p.adj<0.05), and other parameters were set to default values. Genes differentially expressed between cells with high and low TRP scores were considered to be involved in tryptophan metabolism at the single-cell transcriptome level.

### Consensus clustering of tryptophan-related genes

In this study, we used the 14 prognostically relevant tryptophan-related genes described above. We used the “ConsensusClusterPlus” package for consistent clustering (reps = 1000, pItem = 0.8, pFeature = 1, clusterAlg = “pam”), and the optimal number of clusters was evaluated by the cumulative distribution function (CDF) plot and the consensus heatmap with an optimal K-value of 2. We used the “survival” package to evaluate the clinical survival outcomes of LUAD samples in molecular subtypes based on TRPRGs. Principal component analysis (PCA) was used to investigate the distribution patterns of molecular isoforms based on TRPRGs using the R package “ggplot2”. Finally, we used “ggplot2” R to analyze the distribution pattern of molecular subtypes based on TRPRGs. Finally, we used the “pheatmap” R package to visualize the relationship between TRPRGs expression, clinical survival status, and clinicopathological features.

### Enrichment analysis and functional annotation

To further investigate differentially expressed genes (DEGs) between subgroups defined by TRPs, we identified DEGs using the “limma” R package with screening criteria of |logFC|> 1 & p < 0.05 and followed up with further analysis of genes associated with prognosis. To explore the underlying mechanisms of the two tryptophan metabolism subtypes involved in LUAD, we performed gene set enrichment analysis (GSEA) in different clusters constructed based on tryptophan metabolism-related genes. The “h.all.v7.4.Hs.symbols” and “c2.cp.kegg.v7.4.symbols.gmt” gene sets downloaded from MsigDB were used as the reference gene sets, while the “GSVA” package was used to calculate the enrichment scores of the relevant pathways. We calculated the differentially expressed pathways between the two subgroups, where P < 0.05 was considered significant. The gene sets for GSVA and GSEA were downloaded from the Molecular Signatures Database (MSigDB) v7.4 database.

### Characterization of the LUAD immune profile

In this study, the ESTIMATE algorithm was utilized to estimate immune cell abundance between high and low-risk groups using expression data from the TCGA database. The relative proportions of the 22 immune cell types in each tumor tissue were estimated using the CIBERSORT algorithm based on the TPM values of the TCGA-LUAD patients, and samples with P > 0.05 in the results were excluded and the remaining samples were analyzed further (34). In addition, we determined the level of immune cell infiltration in LUAD TME by using the single sample gene set enrichment analysis (ssGSEA) algorithm (35), and unique combinations of characterized genes for each immune cell subtype were obtained from the most recent literature (36, 37). In addition, this study used the TIDE (http://tide.dfci.harvard.edu/) (38) algorithm to calculate immune escape scores between the two subgroups.

### Construction and validation of TRP-related prognostic risk profiles

To explore the prognostic value of LUAD based on TRPRGs, we performed univariate Cox regression analyses (P < 0.0001) and least absolute shrinkage and selection operator (LASSO) regression analyses of DEGs between subgroups (39) and stepwise multifactor Cox regression analyses identified independent characteristic prognostic factors to establish the prognostic profile of LAUD. The tryptophan risk score (TRPRS) was then calculated for each LUAD patient based on the risk coefficients and LUAD expression profiles obtained in the multifactorial Cox regression analysis using the formula: TRPRS = 0.039*DLGAP5 + 0.148*ANLN + 0.083*PTTG1 + 0.118*RHOV + 0.129*FAM83A. Subsequently, we used LUAD patients from the TCGA cohort as the training set and GSE31210, GSE50081, and GSE30219 from the GEO database as the validation set, and categorized the LUAD patients into two groups, low-risk and high-risk, based on the median risk score. Kaplan-Meier survival curves and Log-Rank tests were used to assess whether there was a significant difference in OS between the low-risk and high-risk groups. Finally, we validated the prognostic predictions of the risk model using time-dependent ROC curves to calculate 1-, 3-, and 5-year AUC values in the validation cohort.

### Genomic alteration analysis

Somatic mutation and copy number variation (CNV) profiles were obtained from the TCGA data portal (https://portal.gdc.cancer.gov/). Somatic mutation and CNV (GISTIC output) data were visualized using the R package “maftools” (40). The Significant amplification or deletion of copy number was detected using GISTIC 2.0 with a threshold FDR Q < 0.05.

### Immunotherapy response prediction and independent cohort validation

Tumor mutational burden (TMB), a potential biomarker of immunotherapy response, is calculated based on somatic non-synonymous mutations. In addition, Tumor Immune Dysfunction and Exclusion (TIDE), a computational algorithm that assesses T-cell dysfunction characteristics, can be predicted by immunotherapy response in patients with expression profiles LUAD. and has shown greater efficiency in predicting anti-PD1 or anti-CTLA4 treatment responses (38). In addition, tumor immunophenotype scores, another biomarker of immunotherapy response, were obtained from The Cancer Immunome Atlas (TCIA) and analyzed. The prediction of immunotherapy efficacy was validated using risk modeling using five immunotherapy cohorts: advanced uroepithelial carcinoma, an anti-PD-L1 antibody (IMvigor210 cohort) (41). Melanoma treated with adoptive T-cell therapy (ACT) (GSE100797) (42); anti-CTLA4 and anti-PD1 therapy (GSE91061) (43); Melanoma cohort treated with MAGE-A3 antigen immunotherapy (GSE35640) (44); NSCLC treatment with pembrolizumab, anti-PD-1 antibody (GSE126044) (45); TCGA cohort responses and gains are based on the TIDE algorithm.

### Comprehensive analysis of five prognostic signatures

First, we analyzed the mRNA expression levels of RHOV, PTTG1, FAM83A, DLGAP5, and ANLN from the TCGA and GEO database in LUAD patients and the normal group, and then grouped LUAD patients into high and low groups by the median of the expression values of the transcript levels for survival curve analysis. We then explored the expression differences of the five genes in lung adenocarcinoma at the single-cell level. In addition, scMetabolism, a recently developed computational pipeline for quantifying single-cell metabolism, was applied to visualize and quantify the metabolic diversity of individual cells in each cluster (46). The Metabolic activity was quantified at single-cell resolution by the “scMetabolism” R package, using the “VISION” function and KEGG as the reference gene set. Subsequently, we analyzed staging expression differences in five prognostic signatures and performed correlation analyses with TME and metabolic pathways.

### Drug sensitivity analysis

Drug sensitivity data were obtained from Genomics of Drug Sensitivity in Cancer (GDSC2, https://www.cancerrxgene.org/) by downloading GDSC2 gene expression profiles and corresponding drug response information. Ridge regression models that can be applied to lung adenocarcinoma and sensitivity data for 198 drugs were generated. Lower 50% inhibitory concentration (IC50) values indicate increased sensitivity to compound response. Using the “oncoPredict” R package, we calculated drug sensitivities for the TCGA-LUAD cohort.

### Cell culture and transfection

Human lung adenocarcinoma cell lines A549 and H1299, and human normal lung epithelial cell BEAS-2B were mainly purchased from the cell bank of the Chinese Academy of Sciences (Shanghai, China). We used A549 and H1299 cells for *in vitro* culture experiments in DMEM medium and RPMI 1640 medium (Gibco, ThermoFisher Scientific, United States) supplemented with 10% fetal bovine serum, 1% penicillin and streptomycin (Gibco). Small interfering RNA (siRNA) targeting PTTG1 and interfering RNA control were purchased from Gemma Genetics (Shanghai, China). For transient transfection, A549 and H1299 cells were transfected with siRNA using a transfection reagent (Lipofectamine 2000) for 12 h, followed by functional assays and subsequent experiments.

### Immunohistochemistry

All experiments involving human tissues were performed following the principles of the Declaration of Helsinki and approved by the Institutional Review Board of Guilin Medical University Hospital (No. 2022YJSLL-78). We collected a total of 17 lung tumor tissues (15 lung adenocarcinoma tissues, one case each of squamous lung cancer as well as small cell lung cancer tissues) and 7 peripheral lung tissues from patients with lung adenocarcinoma. Baseline data on the patients were collected in **Supplementary Table 6**. The following primary antibody and antigen recovery protocol was used: PTTG1 (Proteintech, 18040-1-AP, China). Immunohistochemical histologic scoring criteria: Positive cells were defined by the presence of brownish-yellow granules in the cytoplasm and nucleus of the cells. Scoring was based on the intensity of staining of the cells counted under high magnification, with blue color being 0, tan color being 1, and brown color being 2; scoring was also based on the percentage of positive cells, with 0- 10% being 1, 11- 30% being 2, 30- 50% being 3, and greater than 50% being 4. The product of two scores greater than 3 was scored as PTTG1(+). Embedded wax blocks were sliced and deparaffinized with a gradient of xylene and alcohol. After rinsing with water, endogenous peroxidase interference was removed by hydrogen peroxide immersion, followed by antigen repair by adding EDTA in an autoclave. After cooling at room temperature, it was rinsed with PBS buffer, closed by adding serum, and incubated in an incubator at 37 degrees Celsius for 30 min. Primary and secondary antibodies were incubated separately, and then color was developed using DAB and background stained with hematoxylin.

### RT-qPCR and western blotting

RNA was extracted from lung adenocarcinoma cell lines (A549, H1299) that interfered with si-PTTG1 and NC as a control. SYBR Green qPCR mix (Vazyme, China) was used to synthesize cDNA for real-time PCR. protein blotting analysis RIPA lysis buffer (Servicebio, China) containing PMSF (Servicebio, China) was used to collect proteins from A549 and H1299 cells. 10% sodium dodecyl sulfate-polyacrylamide gel electrophoresis (SDS-PAGE) was used to separate the protein samples, and polyvinylidene difluoride (PVDF) membranes (Immobilon-P, Carlsbad, Ireland) were used to transfer the separated proteins. The membrane was blocked for 15 min using a rapid blocking solution and then incubated with primary antibodies: PTTG1 (Proteintech, 18040-1-AP, 1:1,000) and β-actin (Proteintech, 66009-1-Ig, 1:20,000) overnight at 4°C, followed by 2 h of incubation with secondary antibodies.

### Proliferation and clone formation experiments

Cell proliferation and colony formation assay A549 and H1299 cells were cultured in 96-well plates (3,000 cells/well) 24 hours after transfection with PTTG1 siRNA. The proliferative capacity of the treated cells was assayed at 4, 24, 48 and 72 hours. 10% Cell Counting Kit-8 (CCK8) reagent (Bio-sharp, Hefei, China) was added to each plate according to the kit instructions, and the OD450 values were analyzed by an enzyme marker (BioTek, United States). Regarding colony formation experiments, 2000 cells were inoculated in cell culture plates and allowed to grow until visible colonies were formed. Then we fixed the clones with paraformaldehyde for 15 min, stained the clones with 1% crystal violet for 20 min, and counted the number of clones (>50 cells).

### EDU staining experiment

Cell proliferation was detected using a 5-ethynyl-2′-deoxyuridine (EdU) assay (BeyoClick™ EdU-555 Cell Proliferation Detection Kit, Beyotime, China). First, siRNA-transfected LUAD cells were collected and uniformly replanted in glass-bottomed dishes containing 10% FBS (2×10^4^ cells/well). After 24 hours of incubation, 100 μL and 50 mmol/L EdU labeling solution diluted with complete DMEM was then added to each dish and incubated with the cells for 2 hours at 37°C. Experiments were then performed according to the instructions in the kit operation manual. Finally, the images were visualized by fluorescence microscopy.

### Transwell migration experiments

Transwell migration and wound healing assay A549 and H1299 cells were transfected with PTTG1 siRNA for 24 h and cultured in 24-well culture plates with 8 mm pore membrane inserts to measure cell migration capacity. 4 × 10^4^ cells were inoculated in the upper chamber of a transwell with 200 ul of serum-free medium, and 800 μl of medium containing 10% FBS was added to the lower chamber. After 48 h of incubation, cells migrating across the membrane were fixed with paraformaldehyde, stained with 1% crystal violet, and counted under a light microscope (50×).

### Apoptosis assay

A549 or H1299 cells were inoculated in 6-well plates and treated for transfection for 24 h after cell adhesion. Cells were digested with trypsin without ethylenediaminetetraacetic acid (EDTA) and washed with pre-cooled PBS. The collected cells were suspended in 100 μL of binding buffer (1×) and then incubated with 5 μL of Annexin V-FITC and 5 μL of PI for 25 min in the dark (37°C) before adding 400 μL of binding buffer (1×). Finally, the stained cells were measured with a FACSVerse flow cytometer (BD Biosciences, USA) within 1 h after staining and then analyzed with FlowJo software (version 7.6.1; Treestar, USA).

### Statistical analysis

Statistical analysis and plotting were performed using R software (version 4.0.1) and GraphPad software. The Wilcoxon test was used for the test between the two paired groups, categorical variables were compared by Chi-square test or Fisher exact test, and the statistical significance of the cell line experiment was assessed by t-test in GraphPad Prism version 9 software. Differences were considered statistically significant at *p < 0.05, **p < 0.01, ***p < 0.001, ****p < 0.0001.

## Results

### Research Flowchart

**Figure 1:**
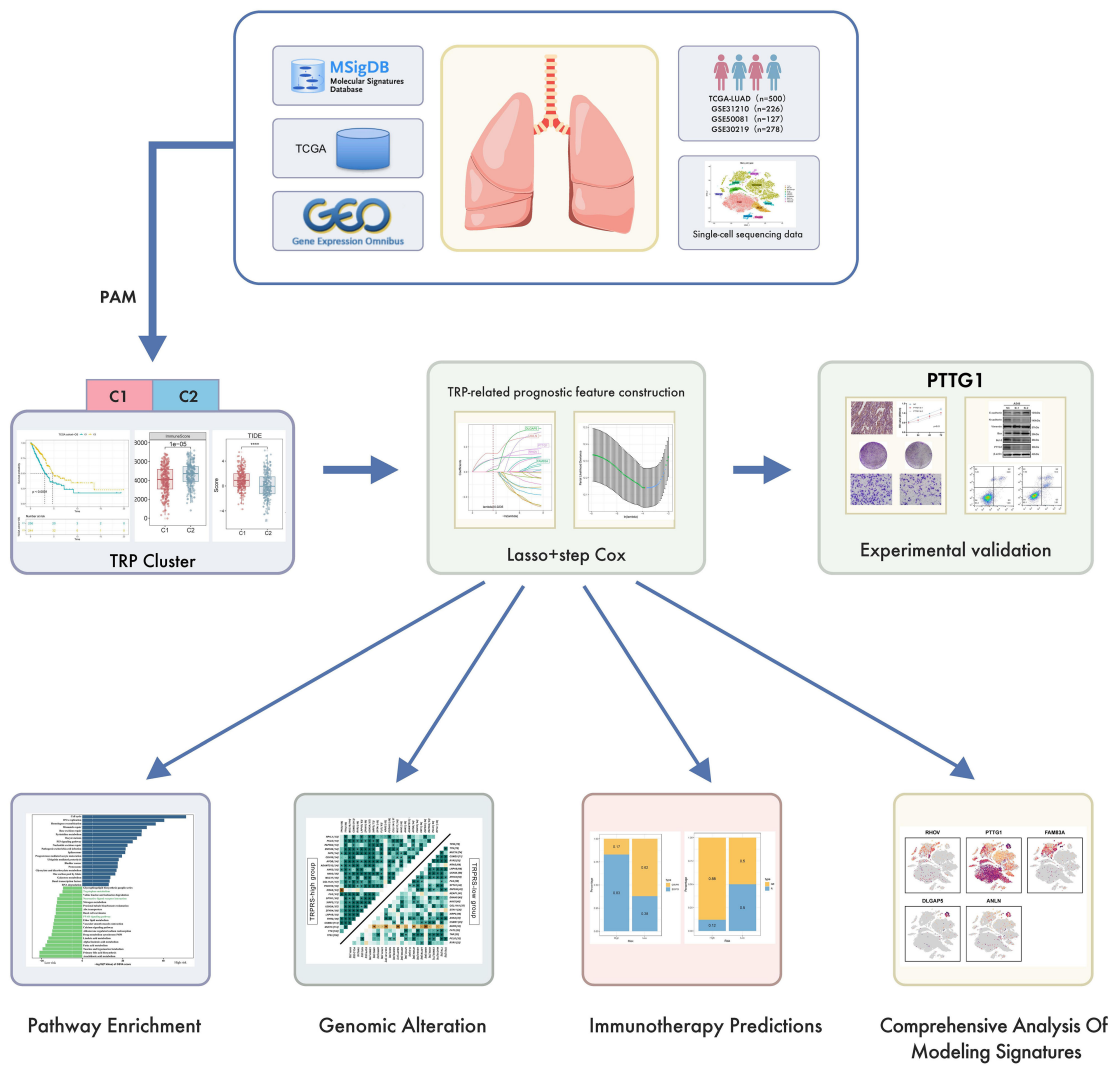
Flowchart of this study.

The flow chart of this study is shown in **Figure 1**. A total of 51 TRPRGs were first obtained from the MSigDB database. Univariate Cox regression analysis showed that 14 of the 51 TRPRGs were associated with OS prognosis in LUAD patients. Therefore, this study used these 14 prognostically relevant TRPRGs for subsequent analysis. Initially, single-cell sequencing data was employed to evaluate the activity of the aforementioned 14 genes, categorizing certain immune cells in individual cells into two expression categories, high and low, and performed difference analysis and pathway analysis on the high and low groups to demonstrate that tryptophan metabolism pathway was significantly up-regulated in the high expression group. Then, consensus clustering using the above 14 genes was performed to categorize LUAD patients into two clusters and predict the survival outcomes of the two subgroups. We performed differential and pathway enrichment analyses of the two subgroups using “limma”, “GSVA” and “GSEA”, and characterized the TME between the subgroups. Next, we performed univariate Cox analysis and lasso analysis on the differential genes to screen five key genes for constructed risk modeling. The high and low-risk groups were characterized by pathway enrichment, genomic alterations, and immunotherapy response prediction, respectively. In addition, we performed a comprehensive analysis of the five prognostic characteristics to determine their correlations with prognosis, clinical features, immunity, and metabolism, and finally, we performed a series of experimental validation of PTTG1, demonstrating that knockdown of PTTG1 reduced the proliferation and migration ability of lung adenocarcinoma cells while increasing apoptosis, and found that the transcription of the key enzyme of tryptophan metabolism, TDO2, was down-regulation after PTTG1 down-regulation (**Figure 1**).

### Genetic variation and expression of prognosis-related TRPRGs in LUAD

**Figure 2:**
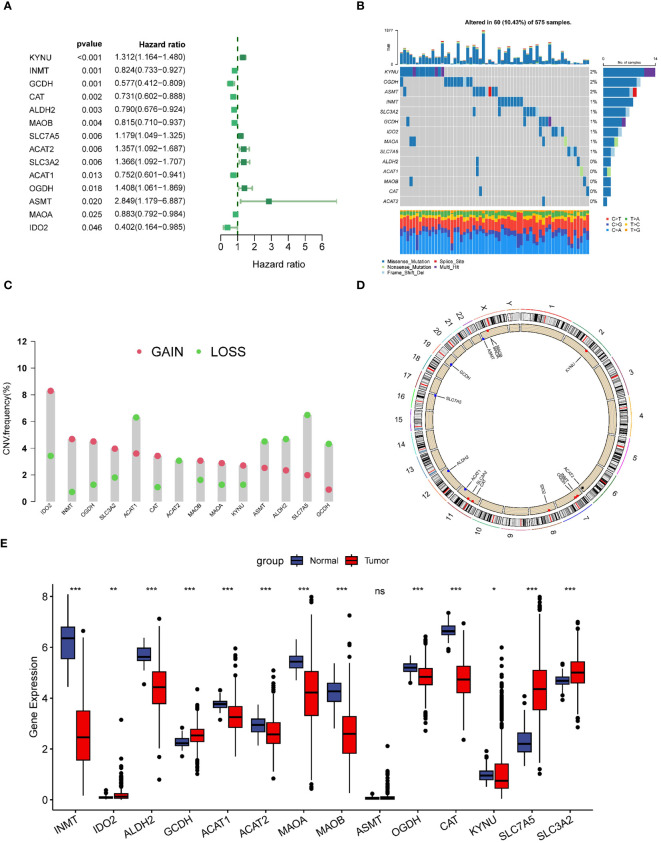
Genetic variation and expression of TRPs in LUAD. **(A)** Forest plot of univariate regression analysis of LUAD patients in the TCGA-LUAD dataset; **(B)** Distribution and mutation frequency of 14 TRPs in the TCGA-LUAD cohort. **(C)** CNV alteration frequencies of TRPs in LUAD, and the height of the bar represent the mutation frequency. **(D)** Location of CNV alterations in TRPs on chromosomes. **(E)** Expression of 14 TRPs genes in LUAD tumors and normal tissues. *p < 0.05. **p < 0.01. ***p < 0.001. ns, p > 0.05.

In this study, we included a total of 51 TRPRGs. In lung adenocarcinoma patients, we used univariate Cox regression analysis to obtain 14 prognostic genes associated with OS, which were demonstrated with a forest plot (**Figure 2A**). In LUAD patients, TRP gene mutations were found in 10.43% (60/575). Among them, KYNU was the gene with the highest mutation rate, followed by OGDH and ASMT (**Figure 2B**). We then examined somatic copy number variation (CNV) in TRPs and found prevalent copy number alterations in 14 TRPs. Among them, IDO2, INMT, OGDH, and SLC3A2 showed extensive CNV amplification and CNV depletion was present in some TRPs (**Figure 2C**). **Figure 2D** demonstrates the location of CNV alterations in tryptophan metabolism-related genes on the chromosome. We further explored the differential expression of tryptophan metabolism-related genes. By comparing the expression levels between LUAD tumors and normal tissues, we found that multiple TRPRGs showed low expression in tumors, except IDO2, GCDH, SLC7A5, and SLA3A2 (**Figure 2E**).

### Features related to tryptophan metabolism in the single-cell transcriptome

**Figure 3:**
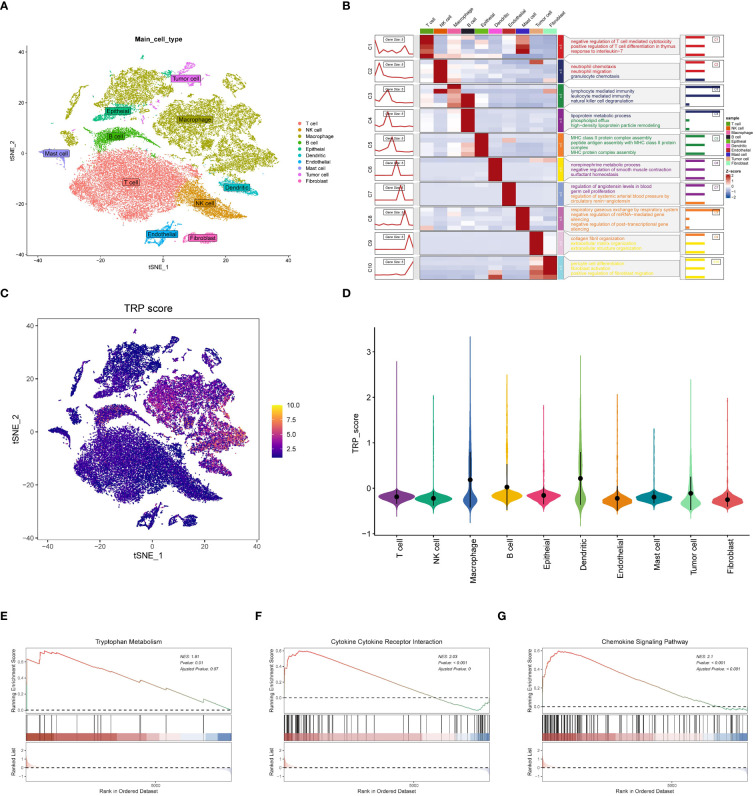
Features related to tryptophan metabolism in the single-cell transcriptome. **(A)** t-SNE plot showing the cell types identified by marker genes. **(B)** Heatmap showing the 5 most important marker genes in each cell cluster. **(C)** Tryptophan metabolism score for each cell; **(D)** Tryptophan metabolism score of different cell types. **(E-G)** Enrichment analysis of GSEA in the high TRP group including tryptophan metabolic pathway **(E)**; cytokine receptor interaction pathway **(F)** and chemokine signaling pathway **(G)**.

We collected single-cell RNA sequencing data from 10 LUAD patients using the single-cell sequencing data from Bischoff et al. Using marker genes for different cell types, we labeled cells into 10 major clusters, i.e., tumor cells, T cells, fibroblasts, macrophages, dendritic cells, mast cells, endothelial cells, epithelial cells, NK cells, and B cells (**Figure 3A**). Enrichment heatmaps showed the marker genes for each cell population (**Figure 3B**). To quantify the activity of tryptophan metabolism (TRP) in different cell types, we used the “AddModuleScore “ function in the Seurat software package to calculate the expression levels of the set of 14 genes associated with TRP in all cells (**Figure 3C**). Among these 10 cell types, we observed significantly elevated TRP activity in macrophages, dendritic cells, and B cells (**Figure 3D**). Based on TRP activity, we classified the cells into high and low TRP groups and identified differentially expressed genes (DEGs) between the two groups for GSEA enrichment analysis. The results showed enrichment to tryptophan metabolism pathway, cytokine receptor interaction, and chemokine signaling pathways in the high TRP group (**Figures 3E-G**), as well as antigen presentation and processing Nod-like receptor signaling pathways (**Supplementary Figures 1A, B**).

### Recognition of TRP-related clusters and altered biological processes

**Figure 4:**
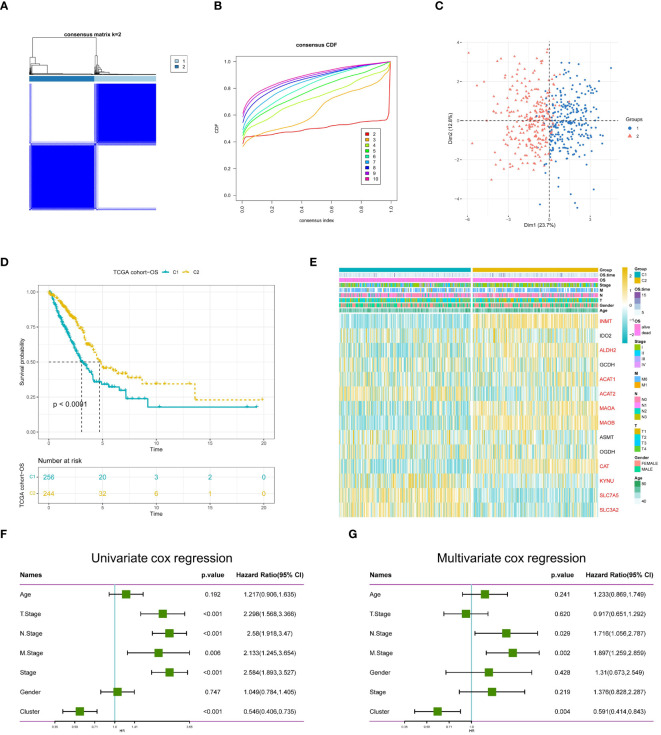
Identification of TRP molecular isoforms. **(A)** Consensus heatmap matrix and correlation region (k = 2) for two clusters **(B)** indicates that the clustering results are best at K = 2. **(C)** PCA analysis indicates significant differences between the two isoforms. **(D)** Survival analysis indicated a better prognosis for C2. **(E)** Differences in clinicopathologic features and TRG expression levels between the two subtypes, with red markers representing differentially expressed genes, p < 0.05. **(F)** Univariate demonstration of clinicopathologic factors and TRP subtypes. **(G)** Multivariate demonstration of clinicopathologic factors and TRP subtypes.

To further explore the profiles and characteristics of 14 tryptophan metabolism-related genes in LUAD, this study applied a consensus clustering algorithm to classify LUAD patients based on the expression of the 14 TRPs. To obtain the optimal number of clusters (k-value), we calculated the consistency coefficient and found that k = 2 was the best choice for classifying the entire cohort into clusters C1 (n = 256) and C2 (n = 244) (**Figures 4A, B**). Principal component analysis (PCA) showed that LUAD patients were well-distributed in both clusters (**Figure 4C**). Kaplan-Meier survival analysis showed that C2 had a superior OS in LUAD (p < 0.0001) (**Figure 4D**). In addition, we obtained consistent results on the GSE30219 cohort (**Supplementary Figures 1C-H**), where we compared the clinicopathologic features and expression of tryptophan metabolism-related genes in the two subtypes. Some TRPs were highly expressed in C2, such as INMT, ALDH2, MAOA, and MAOB, while some TRPRGs, including SLC7A5, SLC3A2, ACAT2, and KYNU, were highly expressed in C1 (**Figure 4E**). We then used univariate and multivariate Cox regression analyses to confirm cluster grouping as an independent prognostic factor for LUAD (**Figures 4F, G**).

**Figure 5:**
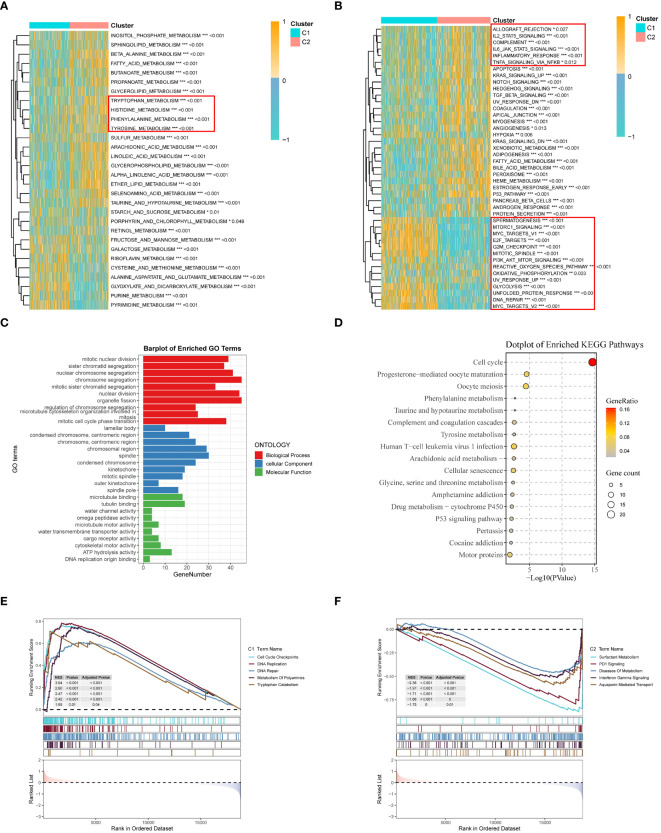
Clinicopathological characterization, enrichment analysis, and mutational landscape of the two TRP clusters. **(A)** GSVA demonstrates the HALLMARK pathway in different subtypes, yellow represents promotion and blue represents suppression; **(B)** GSVA demonstrates the immune part of the pathway in different subtypes; **(C)** GO enrichment analysis of differential genes between the two clusters; **(D)** KEGG enrichment analysis of differential genes; **(E, F)** GSEA enrichment analysis; **(E)** GSEA demonstrates the cancer pathway in C1; **(F)** GSEA demonstrates the immune pathway of C2 subtype. *p < 0.05. **p < 0.01. ***p < 0.001.

To investigate the biological characteristics of the two subtypes, gene set variation analysis (GSVA) results showed that multiple amino acid metabolic pathways (tryptophan, histidine, phenylalanine, and tyrosine metabolism) were significantly enriched in C2 (**Figure 5A**), and the different pathway relationships between the two subtypes were next compared. We performed a GSVA based on the tumor Hallmark gene set to investigate the molecular biological functions of TRP isoforms, and the heatmap demonstrated the pathways with significant differences. The results showed that C1 was significantly enriched in pathways significantly associated with oncogenic activation and highly proliferative features, such as MYC target V1/V2, G2M checkpoint, E2F target PI3K/AKT/mTOR, unfolded protein response, glycolysis, and DNA repair. And C2 is highly expressed in immune pathways, such as IL2/STAT5 signaling pathway, IL6/JAK/STAT3 signaling pathway, allograft rejection, and inflammatory response. Also oncogenic pathways, such as TGFβ signaling pathway, NOTCH signaling pathway, and Hedgehog signaling pathway were highly enriched in C2 (**Figure 5B**).

Next, we also performed functional enrichment analysis of DEGs between subgroups to study the biological behavior of TRP. The results showed that biological processes (BP) indicated the enriched functions of nuclear division and chromosome segregation regulation. Cellular components (CC) showed that TRP was mainly associated with lamellipodia, spindle, and chromosomal regions. For molecular function (MF), it was mainly enriched for microtubule protein binding, microtubule motility activity, and cytoskeleton motility activity (**Figure 5C**). Then, pathway analysis showed that these genes were frequently involved in cell cycle, substance metabolism, viral infection, and cancer-related pathways (**Figure 5D**).

Finally, in the GSEA analysis based on the REACTOME gene set, we observed that the C1 subgroup was significantly enriched for cell cycle and DNA repair-related pathways, and the enrichment of polyamine metabolism and tryptophan catabolic pathways also caught our attention. Polyamine metabolism has been shown to support malignant tumor proliferation and maintain oncogenic phenotype (47, 48) and in addition tryptophan catabolism is thought to be closely associated with immune response and linked to PD-1 blockade (49). PD-1 signaling, interferon gamma signaling, and metabolic disease pathways were further enriched in subgroup C2 (50, 51). The combination of these results suggests that the activation of tryptophan and related metabolism is closely associated with immunosuppression in LUAD patients and that the C2 subgroup has a stronger potential for immunotherapy (52) (**Figures 5E, F**).

### TME characteristics between subgroups

**Figure 6:**
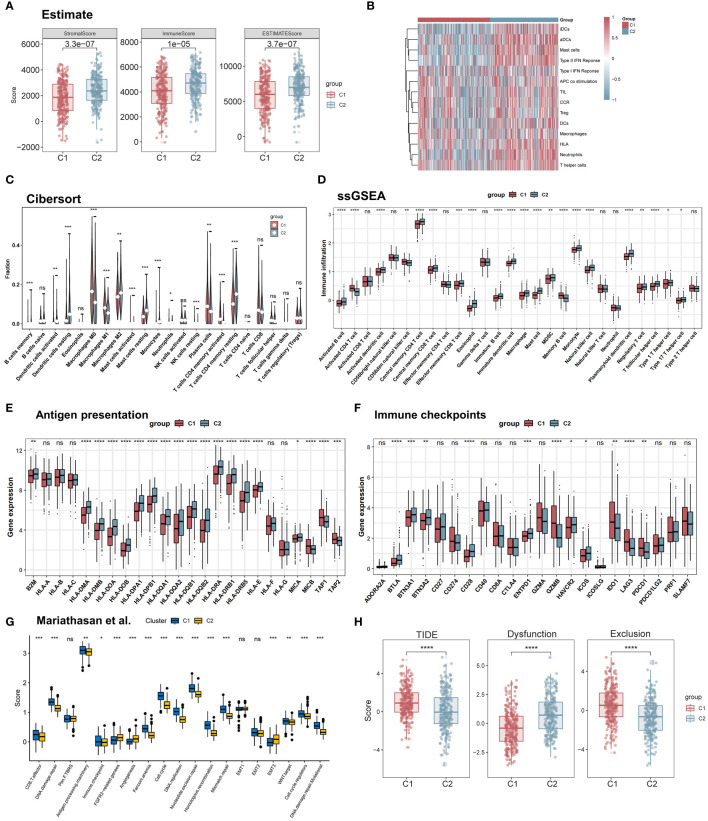
Immune microenvironment of different molecular subtypes. **(A)** Immunity score, ESTIMATE score, and stroma score were used to quantify the different immune status between different subtypes. **(B)** The activity of immune-related pathways differed significantly between C1 and C2 **(C, D)** The abundance of each TME-infiltrating cell type was quantified by the CIBESORT algorithm and the ssGSEA algorithm in different subtype populations. **(E)** Differential expression of HLA molecules in C1 and C2. **(F)** Differential expression of various immune checkpoints in C1 and C2. **(G)** Box line plot demonstrating the differential expression of the Mariathasan et al. gene set in the pathway between the two subtypes; **(H)** TIDE scores of the two subtypes. *p < 0.05. **p < 0.01. ***p < 0.001. ****p < 0.0001. ns, p > 0.05.

To explore the role of TRP in the LUAD tumor microenvironment, first using the ESTIMATE algorithm, we assessed the overall immune infiltration between the two subgroups, which included stromal scores, estimate scores, and immune scores. The C2 subgroup exhibited higher stromal scores and immunity scores (**Figure 6A**). In addition, using the ssGSEA algorithm, we obtained immune cell and associated pathway scores. the C2 subgroup showed significantly greater activity in type I/II interferon response and APC co-stimulation pathways. At the same time, multiple dendritic cells (aDCs, DCs, and iDCs), mast cells, neutrophils, T helper cells, and TILs had a higher infiltration abundance in C2 (**Figure 6B**). Using the CIBERSORT algorithm we found that the C1 subgroup of lung adenocarcinoma patients had higher plasma cells, CD8+ T cells, and M1-type macrophages, and the C2 subgroup had a higher infiltration of M2-type macrophages (**Figure 6C**). The relationship between the two subgroups and 28 immune cells was then assessed by the ssGSEA method. The results showed that the infiltration levels of eosinophils, immature dendritic cells, mast cells, and natural killer cells were significantly higher in the C2 subgroup, while the infiltration levels of activated CD4 T cells, CD56dim natural killer cells, and memory B cells were significantly overexpressed in the C1 subgroup, in addition, we found that MDSC were higher in C2 than in C1, and Activated CD8 T cells and Effector memory CD8 T cells were not significant in the subtypes (**Figure 6D**). Next, we found that most antigen presentation-related genes were significantly highly expressed in C2 between subgroups, so we further investigated the immune checkpoint profiles between the two subgroups, and we found that most of the immune checkpoints were differentially expressed between the two groups including IDO1, LAG3, PDCD1 (PD-1), and HAVCR2 (TIM-3), suggesting that tryptophan metabolism-related isoforms play a potential role in immunotherapy (**Figures 6E, F**). In addition, this feature set was shown to differ between the C1 and C2 subgroups in a variety of biological functions by using a follow-up analysis by Mariathasan et al. Angiogenesis and EMT activity were enhanced in C2, and C1 exhibited higher CD8T cell effector capacity (**Figure 6G**). Finally, we compared the immune escape scores of the two subgroups using the TIDE algorithm, and we found that the TIDE score was higher in C1, i.e., the likelihood of immune escape was higher in the C1 subgroup, and thus the C2 subgroup may have better immunotherapy efficacy (**Figure 6H**). In summary, 500 LUAD patients were classified into two different patterns based on survival-related tryptophan metabolism genes, and the biological processes and immune infiltration characteristics between subgroups showed complexity: C2 had higher immune score and stromal score, as well as high levels of tryptophan metabolism and immune-related pathway activation, and its antitumor activity and immunosuppression showed higher levels; C1 showed poorer prognosis and oncogenic activation and highly proliferative features, along with higher CD8T cell infiltration and immune escape features.

### Construction and validation of TRP-related prognostic features

**Figure 7:**
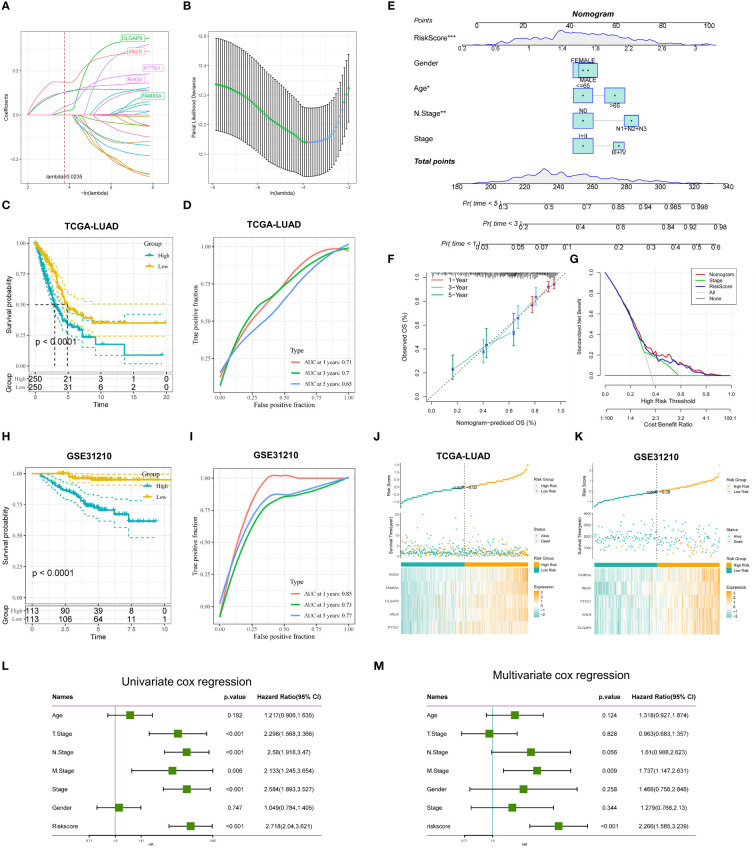
Construction and validation of TRP-related prognostic features. **(A)** Ten-fold cross-validation of parameter selection adjusted by LASSO regression. **(B)** Screening of coefficients under LASSO analysis. A vertical line is plotted at the value selected by 10-fold cross-validation of overall survival. **(C)** KM curves comparing LUAD patients in the TCGA-LUAD high- and low-risk groups **(D)** Time-dependent ROC curve analysis in the TCGA-LUAD cohort. **(E)** Nomogram combining age, gender, N stage, total stage, and risk score. **(F)** Calibration curves of constructed 1-, 3-, and 5-year survival column plots. **(G)** DCA decision curve analysis. **(H)** KM curves demonstrating survival status between high and low-risk groups for GSE31210 **(I)** Time-dependent curves for GSE31210. **(J, K)** Distribution of risk scores and patient survival between low and high-risk groups in the TCGA-LUAD **(J)** cohort, and the GSE31210 **(K)** cohort. **(L)** Univariate and **(M)** multivariate COX regression analyses for characteristics and different clinical features. *p < 0.05. **p < 0.01. ***p < 0.001.

To further assess the impact of DEGs on survival prognosis, we used lasso, univariate, and multivariate regression to screen for five gene signatures with strong prognostic associations. We first used univariate Cox regression analysis (p < 0.001) and found that 25 genes were significantly associated with OS (**Supplementary Table 2**). Next, a 10-fold cross-validated LASSO regression analysis was performed on these 25 genes, and five genes (DLGAP5, ANLN, PTTG1, RHOV, FAM83A) were screened out for further analysis, The tryptophan-associated risk score (TRPRS) for each LUAD patient was calculated according to the following formula: TRPRS = 0.039*DLGAP5 + 0.148*ANLN+0.083*PTTG1 + 0.118*RHOV+0.129*FAM83A (**Figures 7A, B**). We assigned LUAD patients to the high-risk or low-risk group based on the median risk score. Kaplan-Meier analysis showed that patients in the high-risk group had worse OS (p<0.0001; **Figures 7C, H**), and analysis of subject work characteristics (ROC) curves showed that the area under the TRPRS curve (AUC) for the TCGA training set at 1, 3, and 5 years respectively reached 0.71, 0.7, and 0.65, indicating good prognostic diagnostic efficacy, and the validation set GSE31210 was 0.85,0.73,0.77 (**Figures 7D, I**). In addition, to further validate the accuracy and reliability of the five-gene model, we performed supplementary validation in the GSE50081 and GSE30219 cohorts, with 0.83, 0.7, and 0.69 for validation cohort GSE50081, and 0.74, 0.74, and 0.66 for validation cohort GSE30219 (**Supplementary Figures 2A-F**), and the TRP-associated prognostic characteristics showed superior performance. In addition, we further compared our features with two other metabolism-related prognostic models: in Zhou et al.’s study, they modeled the prognosis of lipid metabolism-related mRNAs for LUAD patients, and the ROC curves of the AUC values of the OS-related prognostic subgroups for the 1-, 3-, and 5-year survivals were 0.753, 0.650, and 0.580, respectively (53); in Tang et al.’s study, they constructed a prognostic model for metabolism-related genes predicting response to immunotherapy in lung adenocarcinoma, and the ROC analysis showed that the AUC values of MRG for the TCGA cohort for 1, 2, and 3 years were 0.659, 0.669, and 0.674 (54). Overall, our TRP signature showed better performance in predicting the prognosis of LUAD patients.

To make the TRPRS more suitable for clinical applications, we constructed nomogram based on the TRPRS and clinical features (**Figure 7E**). The calibration curves showed good agreement between the nomogram predictions and the actual observations (**Figure 7F**). Decision curve analysis (DCA) showed that the nomogram had better clinical benefits than other clinical features (**Figure 7G**), and AUC demonstrated the stable predictive ability of the nomogram, which was superior to other clinical features in predicting OS from 1 to 5 years (**Supplementary Figure 1I**). In addition, we constructed heat maps demonstrating the distribution of risk scores, survival status, and risk factors, which showed that there were more deaths and more significant gene expression in the high-risk group (**Figures 7J, K**). To assess whether TRPRS was an independent prognostic factor for LUAD, we performed univariate and multivariate Cox regression analyses of OS in the TCGA-LUAD dataset (**Figures 7L, M**).

### Mutational landscape and drug sensitivity prediction of TRP-related prognostic features

**Figure 8:**
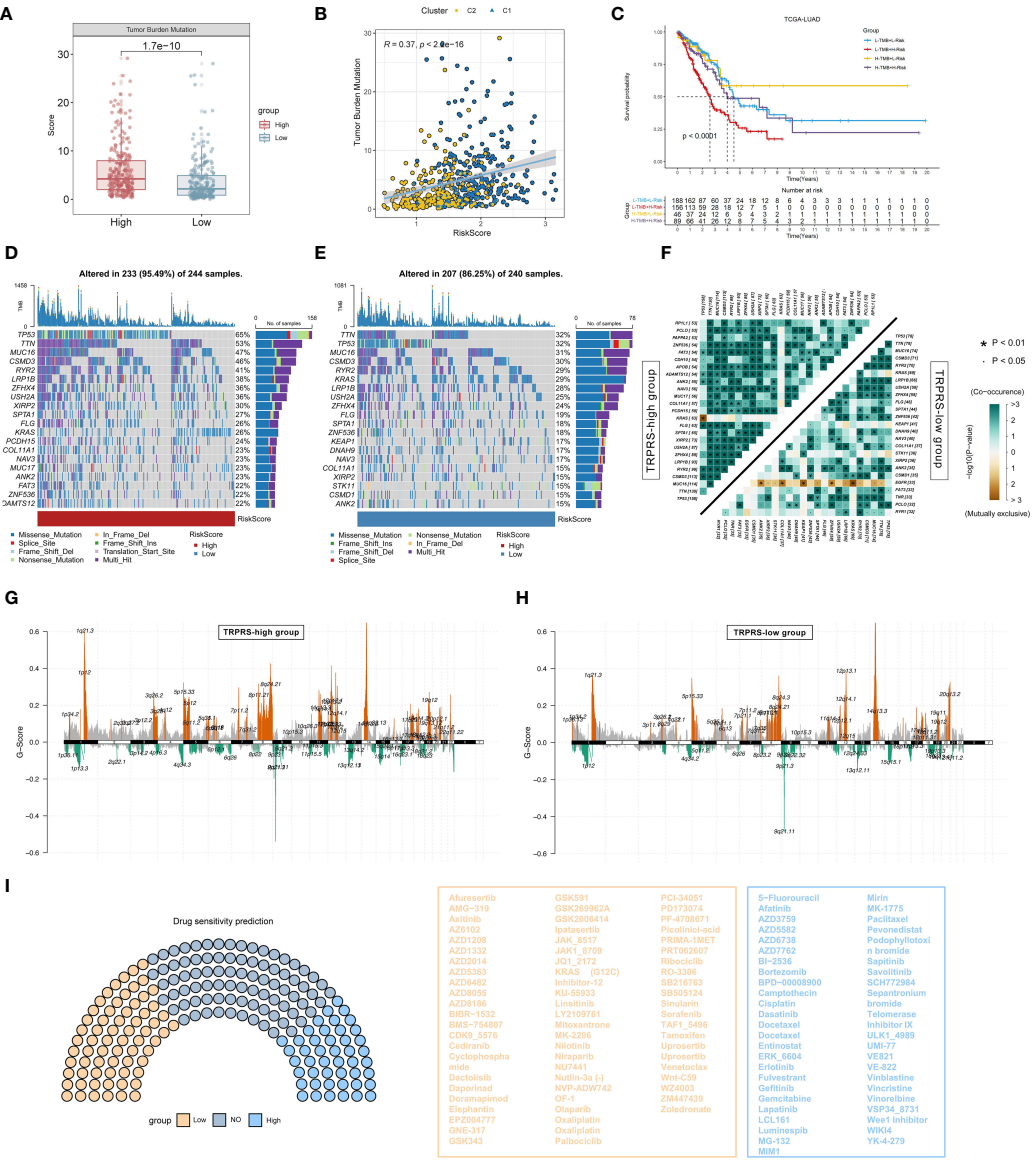
Mutational landscape and drug sensitivity prediction for TRP-related prognostic modeling. **(A)** Box plot graphs demonstrating TMB differences between high and low-risk groups. **(B)** Correlation between TMB and riskscore. **(C)** Kaplan-Meier curve analysis of OS combining TMB score and risk score. **(D)** Waterfall plot of the high-risk group showing mutations. **(E)** Waterfall plot showing mutations in the low-risk group. **(F)** Heatmap showing the relationship between co-occurring and exclusive mutations in the top 25 mutated genes in the high- and low-risk groups. **(G, H)** Copy number variation (SNV) between the two groups **(G)** SNV mutations in the high-risk group; **(H)** SNV mutations in the low-risk group. **(I)** Drug sensitivity analyses between low-and high-risk groups.

First, there was a significant difference in tumor mutation burden (TMB) between high-risk and low-risk scores, with the high-risk score group having a significantly higher tumor mutation burden than the low-risk score group (**Figure 8A**), and then the correlation between TRPRS and TMB was explored, and using Spearman’s correlation analysis, it was found that the risk scores had a significant positive correlation with TMB (R = 0.37, P< 0.001) (**Figure 8B**). After integrating the TMB scores, LUAD patients in TCGA were categorized into four groups. Survival analysis showed that patients with high TMB and low risk had a significant survival advantage, and the low TMB and high-risk groups exhibited a significant survival disadvantage (**Figure 8C**). In the TCGA-LUAD cohort, changes in the distribution of somatic mutations between the low-risk and high-risk groups were investigated (**Figures 8D, E**). Compared with patients with low-risk scores, patients in the high-risk group had significantly higher frequencies of somatic mutations (95.49% vs. 86.25%), especially TP53 (65% vs. 32%), TTN (53% vs. 32%), MUC16 (47% vs. 31%), CSMD3 (47% vs. 30%), RYR2 (41% vs. 29%), LRP1B (38% vs 28%) and ZFHX4 (36% vs 24%). In addition, the association of co-occurring and mutually exclusive mutations in the top 25 mutated genes in the high-risk and low-risk groups was also investigated, and the results showed that the high-risk group (TRPRS-high group) exhibited a higher frequency of co-occurring mutations, and a specific case of EGFR mutually exclusive mutations was observed in the low-risk group (TRPRS-low group) (**Figure 8F**). We subsequently analyzed somatic copy number variation (SCNV) using GISTIC 2.0 (55) that detected significant amplifications and deletions in each risk group, setting the threshold FDR < 0.05. In contrast, we observed more regions altered in the TRPRS high cohort (**Figures 8G, H**). We further performed the drug sensitivity analysis to predict the semi-inhibitory concentrations of 198 chemotherapeutic drugs (**Figure 8I**). Our results showed that 46 drugs had lower semi-inhibitory concentration values (IC50) in the high-risk group, suggesting sensitivity. In addition, patients in the low-risk group were sensitive to 67 drugs. The results suggest that risk scores can be used as a potential predictor of chemotherapy sensitivity, providing a new understanding of tumor treatment and drug resistance prevention.

### Comprehensive analysis of TRPRS with clinical features, enrichment pathways, and immunotherapy

**Figure 9:**
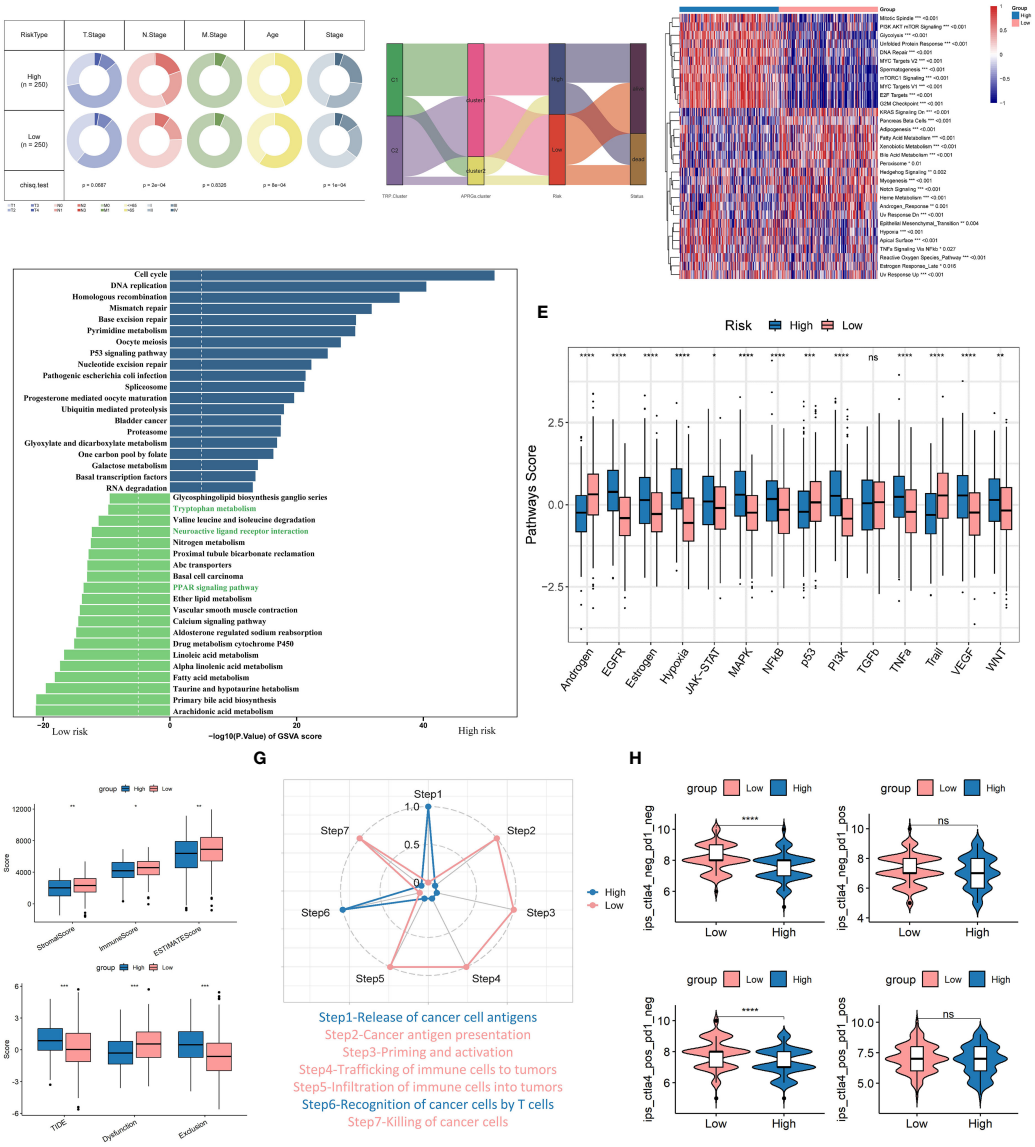
Combined analysis of TRPRS with clinical features, enrichment pathways, and immunotherapy. **(A)** Correlation of low-risk and high-risk groups with clinical features. **(B)** Sankey diagram showing the distribution of LUAD patients. **(C, D)** GSVA analysis of high and low-risk groups. **(E)** Box-and-line plot demonstrating the differences of 14 cancer-related pathways between high- and low-risk groups. **(F)** Differences in the Estimate algorithm and TIDE algorithm between the two groups. **(G)** Differences in the immune seven-part cycle in the high- and low-risk groups. **(H)** Difference of IPS score between the two groups. *p < 0.05. **p < 0.01. ***p < 0.001. ****p < 0.0001. ns, p > 0.05.

We first assessed the correlation between TRPRS and various clinical characteristics. In the TCGA-LUAD dataset, we observed significant differences between the high-risk group and the low-risk group in terms of age, stage, and N stage (p < 0.001, chi-square test) (**Figure 9A**). The Sankey diagram demonstrates the differences in the 2 TRP clusters, 2 APRGs clusters (56), and the distribution of patients diagnosed with LUAD in 2 risk groups (**Figure 9B**). We noted a higher proportion of the C1 subgroup and high-risk in the arginine-associated immune escape cluster (CLUSTER2) in the previous study, and the distribution of patients in the C1 subgroup versus the high-risk group, and the C2 subgroup versus the low-risk group, tended to be consistent. We then performed GSVA enrichment analysis on the high and low-risk groups, and the results showed that various oncogenic pathways were activated in the high-risk group, such as glycolysis, PI3K-AKT-mTOR signaling, DNA repair, MYC signaling, and E2F targets, hypoxia, and significant activation of epithelial-mesenchymal transition (EMT) pathway. In addition, fatty acid metabolism, bile acid metabolism, and heme metabolism were significantly upregulated in the low-risk group (**Figure 9C**). In addition, we performed GSEA enrichment analysis to identify signaling pathways that were differentially activated between TRPRS high- and low-risk group phenotypes (**Supplementary Figures 2G, H**). The results showed that samples from the high-risk group were significantly enriched for cancer-related signaling pathways such as cell cycle, DNA replication, mismatch repair, and P53 signaling pathway; and the low-risk group was enriched for a variety of metabolism-related pathways such as butyrate metabolism, primary bile acid biosynthesis, and metabolic pathways such as leucine and isoleucine degradation. **Figure 9D** demonstrated the top 20 enriched pathways between each risk group, and the high-risk group showed stronger activity in pathways related to Cell cycle, Mismatch repair, and P53 signaling pathway, while the low-risk group showed stronger activity in metabolism-related pathways such as Tryptophan metabolism and Fatty acid metabolism. In addition, previous studies have shown that tryptophan metabolites of the kynurenine (Kyn) pathway (KP) exhibit distinct neural activity (24), whereas the activities of Neuroactive ligand-receptor interaction and PPAR signaling pathway were significantly up-regulated in the low-risk group, which is in line with previous studies. The activity scores of 14 cancer-related pathways were also analyzed, and the results showed that patients in the high-risk group had significantly higher scores of EGFR, hypoxia, and other characteristics (**Figure 9E**), suggesting that TRPRS is closely related to cancer-related biological processes and metabolic pathways. In addition, the Estimate algorithm and TIDE algorithm also obtained similar results with TRP clusters (**Figure 9F**). In addition, we further analyzed the differential abundance of immune cells and immune functions to characterize the TME landscape. In the low-risk group, the presence of various immune cells involved in antigen presentation, processing, and tumor killing was at higher levels, such as aDCs, B cells, DCs, iDCs, T helper cells, and TILs. accordingly, the low-risk group also exhibited active antigen recognition, processing, and presentation signaling and antitumor effects, including APC co-stimulation, HLA, and type II IFN responses (**Supplementary Figure 2I**). The cancer immune cycle was divided into seven sequential processes, and we further assessed the anticancer immune function of the seven-step cancer immune cycle between the high- and low-risk groups by TIP. While the high-risk group presented high activity at step 1 (antigen release by tumor cells) and step 6 (tumor cell recognition by T cells), the low-risk group showed high activity at multiple other steps (**Figure 9G**). In addition, correlation analysis of risk scores supported these results; risk scores were positively correlated with multiple signals such as mismatch repair, cell cycle, DNA replication, base excision repair, and viral oncogenic effects (**Supplementary Figure 2J**). Finally, the low-risk group exhibited higher immunophenotypic scores (IPS), which suggests that low-risk-scoring patients may be more sensitive to immunotherapy (**Figure 9H**).

### TRPRS predicts immunotherapy response

**Figure 10:**
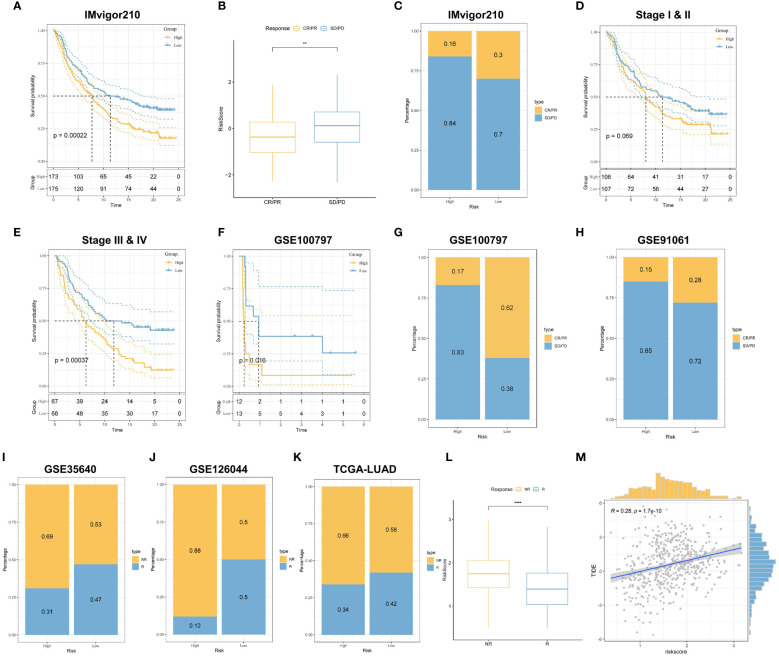
TRPRS predicts immunotherapy response. **(A)** Survival curves for the HR and LR groups in the IMvigor210 cohort. **(B)** Box line plots depicting the difference in risk scores between CR/PR patients and SD/PD patients in the IMvigor210 cohort. **(C)** The proportion of CR/PR or SD/PD patients receiving immunotherapy in the high and low-risk groups of the IMvigor210 cohort. **(D, E)** km curves for the high and low-risk groups of the IMvigor210 staging. **(D)** Stage I-II **(E)** Stage III-IV. **(F)** Survival curves for HR and LR in the GSE100797 cohort. **(G, H)** The proportion of patients with CR/PR or SD/PD receiving immunotherapy in the high and low-risk groups. **(G)** GSE100797; **(H)** GSE91061. **(I-K)** The proportion of patients with R or NR receiving immunotherapy in the high and low-risk groups. **(I)** GSE35640; **(J)** GSE126044; **(K)** TCGA-LUAD. **(L)** Box line plots depicting the difference in risk scores between R and NR patients in the TCGA-LUAD cohort. **(M)** Correlation between TIDE score and risk score. **p < 0.01. ****p < 0.0001.

To further assess the accuracy of risk scores for predicting response to immunotherapy, we selected multiple independent published immunotherapy cohorts for validation. First, we analyzed a cohort of uroepithelial cancers treated with anti-PD-L1 (IMvigor210), and the low-risk group had a significant survival advantage compared with the high-risk group (**Figure 10A**). Also, patients with low-risk scores were more sensitive to immunotherapy (**Figures 10B, C**). In addition, stronger predictive ability was demonstrated in Stage III & IV patients (**Figures 10D, E**). In addition, SubMap analysis was used to assess the response to anti-PD-1 immunotherapy in lung adenocarcinoma patients in high and low-risk groups. The results showed that the low-risk score predicted complete and partial responses (CR, PR) to anti-PD-1 immunotherapy, while the high-risk score predicted resistance (SD) to anti-PD-1 immunotherapy (**Supplementary Figure 3A**). Next, in the melanoma cohort treated with adoptive T-cell therapy (ACT) (GSE100797), the low-risk score also had a strong ability to predict prognosis and immunotherapy benefits (**Figures 10F, G**). Then in the melanoma cohort treated with anti-CTLA4 and anti-PD1 (GSE91061), the melanoma cohort immunotherapeutically treated with the MAGE-A3 antigen (GSE35640), and the NSCLC cohort treated with nilumab (anti-PD-1 antibody) (GSE126044), we found that the low-risk group still showed better immune response, i.e., the high-risk group had less benefit for immunotherapy (**Figures 10H-J**). In the TCGA cohort, we used the TIDE algorithm to predict patients’ responses to anti-PD1 and anti-CTLA4 treatments, and the results showed that the low-risk group was more sensitive to immunotherapy, and TRPRS was significantly positively correlated with the TIDE score (**Figures 10K-M**). Overall, the relevant prognostic features constructed based on tryptophan metabolism were effective in predicting the prognosis and response to immunotherapy in patients with LUAD.

### A comprehensive analysis of five tryptophan-related prognostic features

**Figure 11:**
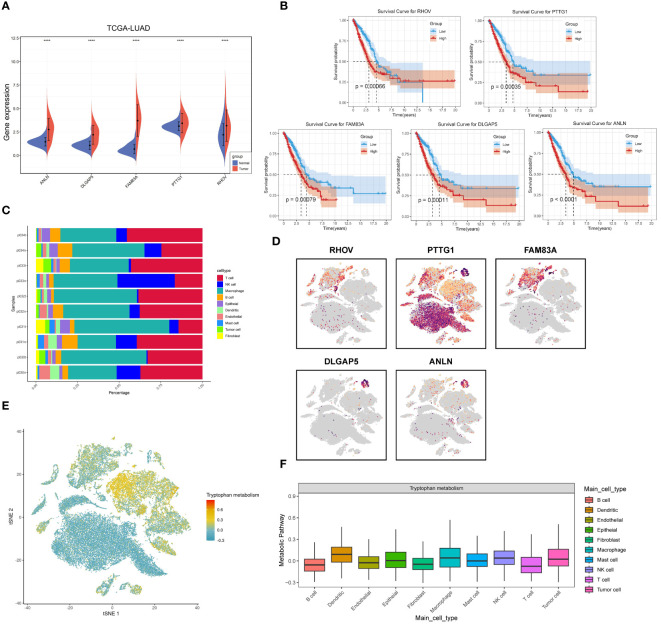
Comprehensive analysis of five tryptophan-related prognostic signatures. **(A)** Violin plots demonstrating the expression of the five prognostic features in cancer and paracancer. **(B)** KM survival curves for the TRPRS gene. **(C)** Single cell occupancy graph. **(D)** Expression level of TRPRS gene in single cells. **(E)** TSNE plot demonstrating tryptophan metabolism levels in single cells. **(F)** Distribution of tryptophan metabolism scores in different cell types. ****p < 0.0001.

First, we compared the differential expression of five tryptophan-associated signatures (ANLN, DLGAP5, FAM83A, PTTG1, RHOV) in tumors versus normal tissues in LUAD (**Figure 11A**), with concordant results in the GSE31210 cohort (**Supplementary Figure 3B**). FAM83A has been shown to correlate with prognosis and immune infiltration in several previous studies and is closely associated with oncogenic phenotype. and is strongly associated with oncogenic phenotypes (57) and is also involved in the construction of multiple LUAD prognostic characteristics (58, 59). FAM83A was significantly overexpressed in lung adenocarcinoma tissues and showed strong diagnostic ability (**Supplementary Figure 3C**). We then performed survival analysis of individual genes (**Figure 11B**), which showed that all five genes demonstrated strong predictive potential, with ANLN showing the most excellent predictive ability (P < 0.0001). We then continued to analyze the expression differences of the five genes at the single-cell level, and **Figure 11C** demonstrates the immune cell and tumor cell occupancy of the 10 selected LUAD sequencing samples, with PTTG1 showing widespread expression in macrophages and T cells, while the other genes were highly expressed in only some macrophages (**Figure 11D**). This may represent a specific role of PTTG1 in the tumor microenvironment. In addition, we calculated the metabolic differences between different immune cells and tumor cells by the scMetabolism algorithm. The results showed that the level of tryptophan metabolism was higher in dendritic cells, macrophages, and NK cells (**Figures 11E, F**).

**Figure 12:**
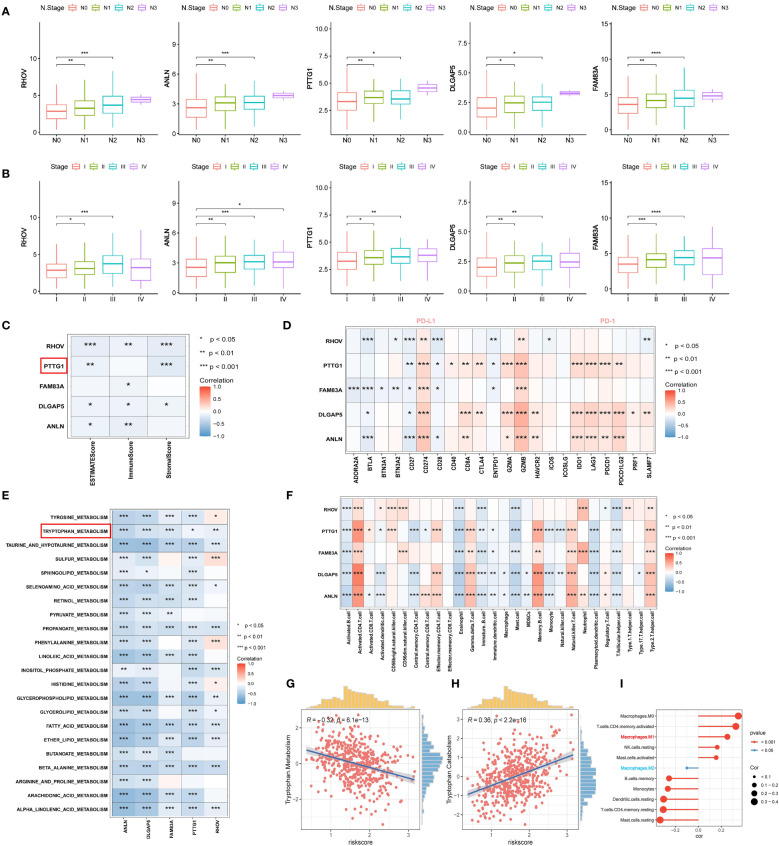
Correlation analysis of five tryptophan-related prognostic features. **(A)** Box plot depicting the correlation of five prognostic features expression with N stage. **(B)** Box plot depicting the correlation of five prognostic features expression with pathologic stage. **(C)** Correlation of five prognostic features with TME score. **(D)** Correlation of five prognostic features with immune checkpoints. **(E)** Correlation of five prognostic features with metabolic pathway score. **(F)** correlation between five prognostic features and 28 specific immune cells. **(G, H)** Correlation of TRPRS with tryptophan metabolism-related pathways. **(I)** Correlation between TRPRS and immune cells. *p < 0.05. **p < 0.01. ***p < 0.001. ****p < 0.0001.

Next, we proceeded to explore the relationship between the expression of RHOV, ANLN, PTTG1, DLGAP5, and FAM83A with pathological stage and lymph node metastasis, and the results showed that the transcriptional expression of the five prognostic features progressively increased with the stage (**Figures 12A, B**). Next, we assessed the correlation between the TME score and the five prognostic features and found that all of them tended to be negatively correlated with the TME score and that PTTG1 was significantly negatively correlated with the stromal score (p<0.0001), and the immune score did not have a significant association (**Figure 12C**). In addition, we explored the correlation of the five prognostic features with the immune checkpoints. It was clear that PTTG1, DLGAP5, and ANLN were positively correlated with most of the immune checkpoints (PD-1, PD-L1, LAG3, CTLA4), whereas RHOV and FAM83A had similar trends (**Figure 12D**). Then, the correlations between the five prognostic features and multiple metabolic pathways were explored, and we found that not only was there a significant negative correlation with tryptophan metabolism, but all of them were negatively correlated with multiple metabolic pathways such as taurine and taurine metabolism, propionate metabolism, fatty acid metabolism, and β-alanine metabolism (**Figure 12E**). This may represent that the relevant prognostic features based on tryptophan constructs may affect the prognosis and immunotherapeutic response of LUAD by influencing multiple metabolic pathways. We further investigated the relationship between the five prognostic features and immune-infiltrating cells. We observed a consistent trend for all five prognostic features to be significantly positively associated with activated CD4 T cells and type II helper T cells. and significantly negatively associated with follicular helper T cells, which contribute to CD8-dependent antitumor immunity and anti-PD-L1 efficacy (60) (**Figure 12F**). In addition, based on the correlation analysis between risk score and tryptophan metabolism ssGSEA score, tryptophan metabolism showed a significant negative correlation with TRPRS (R=0.32, p=6.1e-13), while tryptophan catabolism exhibited a significant positive correlation (R=0.36, p<2.2e-16) (**Supplementary Figure 3D**) (**Figures 12G, H**). Based on the Cibersort algorithm, we found that TRPRS was positively correlated with M1-type macrophages and negatively correlated with M2-type macrophages. And dendritic cells resting and memory CD4T cells resting were significantly negatively correlated with TRPRS (**Figure 12I**).

### PTTG1 promotes lung adenocarcinoma progression and affects tryptophan-related gene expression

**Figure 13:**
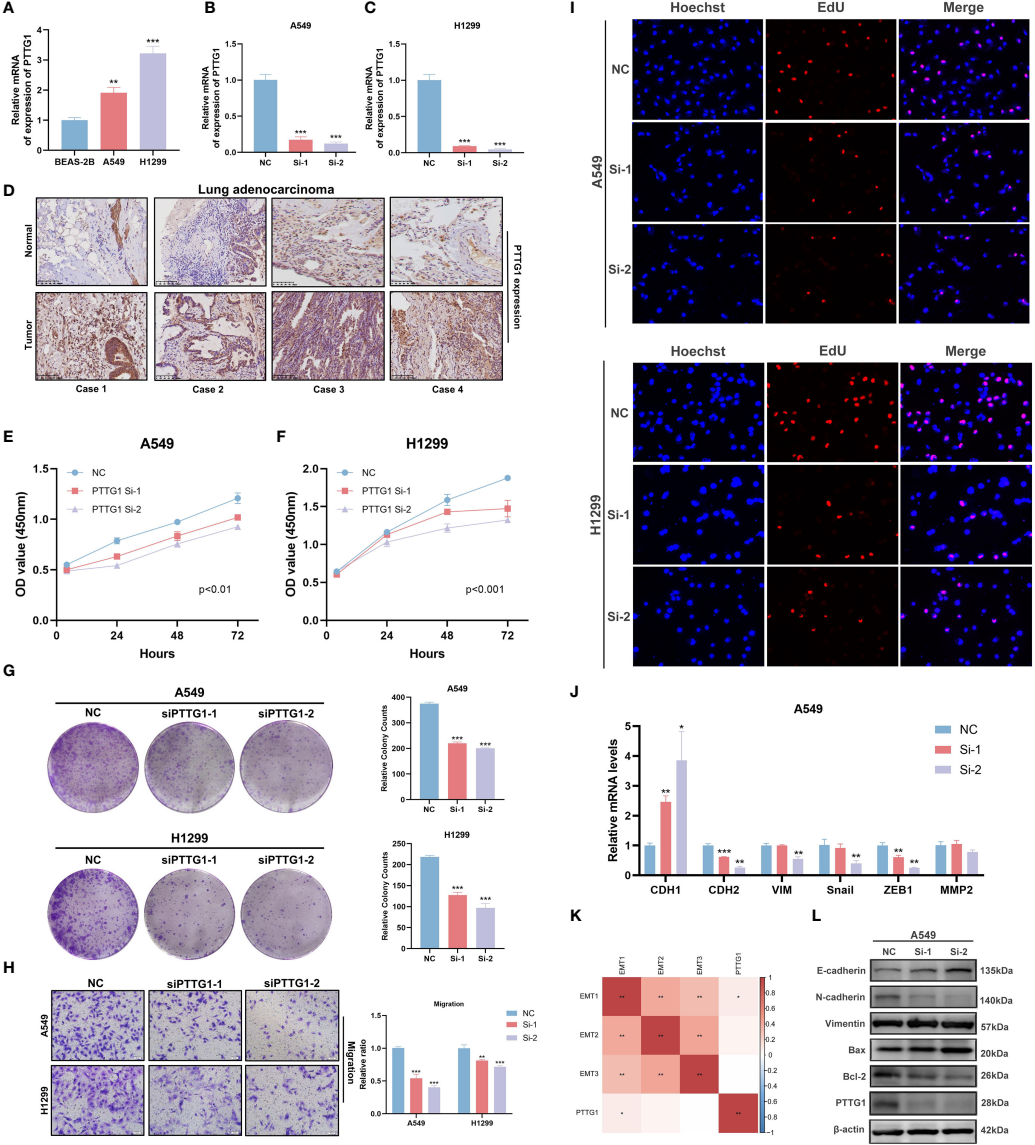
PTTG1 promotes lung adenocarcinoma progression and affects tryptophan metabolism. **(A)** RT-qPCR to verify PTTG1 expression. **(B, C)** RT-qPCR screening for suitable interferences. **(D)** Representative image of IHC (20x). **(E, F)** CCK8 assay. **(E)** A549, **(F)** H1299. **(G)** Clonal spots of A549 and H1299. **(H)** Transwell migration assay. **(I)** EDU staining assay. **(J)** RT-qPCR to verify the effect of knockdown of PTTG1 on key EMT genes from mRNA expression level. **(K)** Correlation analysis of EMT characteristics with PTTG1. **(L)** Western blot verified the effect of knockdown of PTTG1 on EMT and apoptosis markers from the protein level. *p < 0.05. **p < 0.01. ***p < 0.001.

**Figure 14:**
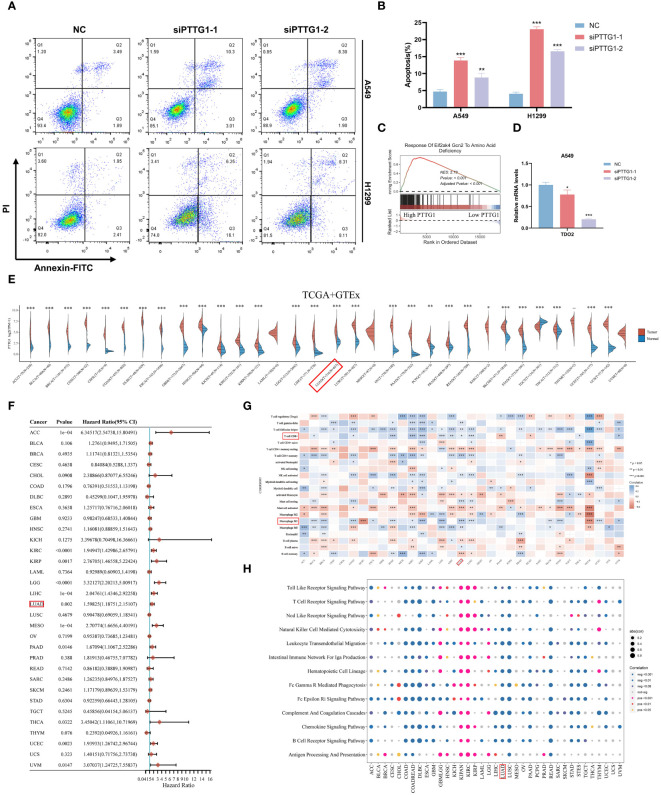
PTTG1 promotes lung adenocarcinoma progression and affects tryptophan metabolism. **(A, B)** Flow cytometry demonstrating knockdown of PTTG1 on apoptosis rate of LUAD cells. **(C)** GSEA analysis showing the correlation of PTTG1 expression with the gene set of GCN2 in response to amino acid deficiency **(D)** Effect of knockdown of PTTG1 on tryptophan key genes. **(E)** Box line plot demonstrating the expression level of PTTG1 in pan-cancer. **(F)** Forest plot demonstrating the prognosis of PTTG1 in pan-cancer. **(G)** Immunological infiltration of PTTG1 in pan-cancer. **(H)** GSEA enrichment analysis demonstrating the pathway of PTTG1 in pan-cancer. *p < 0.05. **p < 0.01. ***p < 0.001.

In the above analysis, we identified a specific role for PTTG1 in the TME, and that has not been fully investigated in LUAD. Next, we performed a series of cell experiments on lung adenocarcinoma. Cell line RT-qPCR results showed that PTTG1 was overexpressed in LUAD cells (A549 and H1299) compared to control cells (**Figure 13A**). The knockdown effect of PTTG1 is shown in **Figures 13B, C**. Interference 2 exhibited higher knockdown efficiency. We performed PTTG1 immunohistochemical staining of lung cancer tissues and peripheral lung tissues of 17 samples from the Guilin lung cancer cohort. The results showed that the lung cancer tissues (lung adenocarcinoma, lung squamous carcinoma, and small cell lung cancer) were all (+) and the surrounding lung tissues were (-) (**Figure 13D**) (**Supplementary Figure 3E**). Some of the lung adenocarcinoma tissues were also subjected to Ki-67 and PD-1 for verification of protein level expression. We found that the degree of PTTG1 (+) positivity correlated with Ki-67 (+) and PD-1 (+) (**Supplementary Figure 3F**). suggesting a correlation between PTTG1 and tumor tissue proliferation and PD-1 therapeutic targets in LUAD patients. CCK8 assay showed that inhibition of PTTG1 may significantly inhibit the proliferative capacity of LUAD cells, and the inhibitory effect of Interference 2 was even more significant (**Figures 13E, F**). The same trend was verified by colony formation assay (**Figure 13G**). The results of the Transwell assay showed that knockdown of PTTG1 significantly inhibited the migration ability of LUAD cells (**Figure 13H**). In addition, we observed a lower percentage of EdU-positive cells in PTTG1 knockdown cells (**Figure 13I**). To further assess the role of PTTG1 in EMT, we examined the mRNA levels of EMT marker genes, which showed elevated E-cadherin mRNA levels and significantly lower mRNA levels of mesenchymal genes (N-cadherin and Vimentin) in A549-interacting cells compared to A549 control cells, as well as a significant reduction in the mRNA levels of the EMT-related transcription factors Snail, ZEB1 and MMP2 were also attenuated in A549 cells with knockdown of PTTG1 (**Figure 13J**). In the TCGA cohort, we found that PTTG1 gene expression showed a significant positive correlation with EMT features constructed by Mariathasan et al (**Figure 13K**). Meanwhile, knockdown of PTTG1 resulted in increased levels of E-cadherin protein, decreased levels of N-cadherin protein, and no significant changes in Vimentin protein levels. In addition, the pro-apoptotic protein Bax was upregulated, and the anti-apoptotic protein Bcl-2 was downregulated. (**Figure 13L**). Flow cytometry results showed that knockdown of PTTG1 significantly increased the apoptosis rate of LUAD cells (**Figures 14A, B**). GSEA showed that high PTTG1 levels were positively correlated with the pathway of cellular response to amino acid starvation in the TCGA-LUAD dataset (**Figure 14C**). RT-qPCR results demonstrated that knockdown of PTTG1 resulted in a significant downregulation of the key tryptophan regulator, TDO2, which may represent a certain degree of PTTG1 influencing tryptophan metabolic processes (**Figure 14D**). Then, we analyzed the expression of PTTG1 in TCGA pan-cancer. The results showed that PTTG1 was highly expressed in 27 tumors and lowly expressed only in TGCT. As in CESC, OV showed significant overexpression (**Figure 14E**). Univariate Cox regression analysis showed that PTTG1 was expressed as a risk factor in most of the tumors, which was a significant risk factor for patients with ACC, KIRC, KIRP, LGG, LIHC, LUAD, and MESO (**Figure 14F**). To explore the relationship between PTTG1 expression and immune cell infiltration, we performed an immune correlation analysis using the Cibersort algorithm. In LUAD, the major anti-tumor immune cells: both CD8T cells and M1-type macrophages showed a significant negative correlation, as well as a consistent trend in KIRC, KIRP, and LGG tumors (**Figure 14G**). This represents a significant immunosuppressive role of PTTG1 in TME. In addition, we analyzed the single-cell sequencing data on the overall characterization of T cells by Guo et al. (61), we found that PTTG1 was highly expressed in CD4_CTLA4 and CD8_LAYN cell subsets, which may suggest that high PTTG1 expression is highly correlated with immunosuppression of tumor-infiltrating Tregs and depleted CD8+ T cell formation (**Supplementary Figures 3G-I**). Finally, GSEA showed that many immune-related pathways, such as the T-cell receptor signaling pathway and the chemokine signaling pathway, were enriched in patients with low PTTG1 expression in most of the cancer types (**Figure 14H**).

## Discussion

Although various anticancer strategies such as surgery, radiotherapy, and targeted therapy are available for the treatment of LUAD, there is an urgent need for effective strategies to cure or control LUAD, especially in patients with advanced LUAD. Immune checkpoint inhibitors (ICIs) have significant advantages in terms of efficacy and safety, bringing new hope for NSCLC treatment (62). Tumor immune escape evades recognition and attack by the immune system through multiple mechanisms (63). The immune escape mechanism of tumors plays an important role in the treatment of tumors, especially in immunotherapy (64). Tumors have various immune escape mechanisms to avoid the monitoring, recognition, and attack of the immune system, such as loss or alteration of tumor antigenicity, weakening of tumor immunogenicity, non-immune-mediated expression of tumor PD-L1, and suppression of immune cell function (65). Immune escape mechanisms help explain the intrinsic and acquired resistance of NSCLC to immunotherapy targeting immune checkpoints (66).

Tryptophan metabolism is directly or indirectly regulated by gut microorganisms, and its metabolites have immune, metabolic, and neuromodulatory functions, and have become therapeutic targets for various diseases (67, 68). Its metabolites have immune, metabolic and neuromodulatory functions and have become therapeutic targets for various diseases. Its main metabolic pathway is the kynurenine pathway (KP), catalyzed by indoleamine 2,3-dioxygenases (IDO1 and IDO2) and tryptophan 2,3-dioxygenase (TDO2). Immune cells are involved in the degradation of tryptophan to kynurenine: first, tryptophan metabolism depletes tryptophan from the local microenvironment and inhibits T cell proliferation and activity (69). Second, by producing kynurenine, it inhibits the proliferation and activity of T cells and natural killer cells and promotes the differentiation of regulatory T cells (70). However, tryptophan metabolism has not been adequately studied in lung adenocarcinoma and its association with immunotherapeutic efficacy has not been addressed.

In this study, we revealed the molecular features and TME landscape of tryptophan metabolism in lung adenocarcinomas through a comprehensive characterization of tryptophan-related genes, with 14 prognostically relevant TRPRGs clinically grouped into two clusters exhibiting different OS and immune profiles. Notably, the C1 subtype had poor OS and dendritic cell infiltration and showed overall strong tumor proliferation and malignant phenotype. In contrast, the C2 subtype exhibited good OS and immune infiltration with a low TIDE score and activation of immune response pathways such as interferon gamma, but C2 also had high levels of infiltration of M2-type macrophages, MDSC, and regulatory T cells, demonstrating a high level of immunosuppression. Next, we screened TRP-related prognostic features and constructed a stable and reliable TRP-related prognostic signature including five TRP-related prognostic signatures (ANLN, DLGAP5, FAM83A, PTTG1, and RHOV.) The TRPRS not only has a strong predictive ability in prognosis but also may possess strong predictive efficacy for LUAD immunotherapy response. In TRPRS-based risk grouping, we found a high degree of concordance between patient stratification based on TRP-associated clusters and high- and low-risk groups, with the low-risk group mainly exhibiting better OS, high immunogenicity, activation of tryptophan metabolism, and lower TMB. Follow-up analyses of the modeled genes also demonstrated that TRPRS was significantly associated with TME and multiple metabolic processes. In addition, we explored the relationship between TRP-related prognostic features and TME and revealed strong correlations between ANLN, DLGAP5, FAM83A, PTTG1, and RHOV with tumor immune infiltration, immune checkpoints, and substance metabolism pathways. The role of PTTG1 in lung adenocarcinoma is unknown, which prompted us to explore the role of PTTG1 as a potential prognostic biomarker for lung adenocarcinoma. In follow-up experiments, we found that PTTG1 promoted the proliferation, migration, and EMT of lung adenocarcinoma cells, as well as inhibited apoptosis of lung adenocarcinoma cells. We also observed a down-regulation of the transcriptional level of TDO2 in A549 cells, suggesting that PTTG1 may affect the level of tryptophan metabolism in lung adenocarcinoma cells. Interestingly, PTTG1, DLGAP5, and ANLN were also included in the metabolically relevant prognostic and immunotherapeutic profiles of lung adenocarcinoma in a recent study (71), suggesting that the relevant prognostic profiles that we constructed do indeed reflect the metabolic profiles of lung adenocarcinoma at the transcriptional level to some extent. However, PTTG1 at the metabolic level has only been reported in hepatocellular carcinoma (27), and its specific mechanism in lung adenocarcinoma needs to be further investigated. Finally, by analyzing the Pan-TCGA dataset, we identified that PTTG1 was significantly up-regulated in a variety of cancers and was significantly associated with poor prognosis; and that high PTTG1 expression was associated with poor infiltration of CD8 T cells and M1-type macrophages in the majority of cancers.

Interestingly, we found a potential link between arginine and tryptophan metabolism during previous studies. In TME, arginine and polyamines can be taken up by dendritic cells, thereby increasing intracellular polyamine content. This induces IDO1 expression and leads to an immunosuppressive phenotype, and Arg1 and IDO1 may be closely related to tumor immunotherapy (72). We observed a correlation between arginine metabolism and immune escape in our previous study and that dendritic cell deletion and impaired T-cell activation were the main features of Cluster 2, which showed similar immune features in the tryptophan-associated cluster. In addition, when KRAS mutations were associated with STK11/LKB1 deficiency, tumors exhibited a “cold” phenotype and were associated with reduced PD-L1 expression (73). This is consistent with the results of the arginine-related cluster. In the tryptophan-associated cluster, we found higher levels of memory CD8T cell infiltration and higher EMT scores in Cluster 2, which may represent that Cluster 2 is closer to an immunosuppressed or immune-excluded phenotype and may reverse its immunosuppression by high levels of dendritic cell and TIL infiltration. In addition, we noted that tryptophan metabolic activity, as quantified by the scMetabolism algorithm, was significantly higher in macrophages, while PTTG1 was also highly expressed in macrophages. In a previous study, Hezaveh et al. found that tryptophan-derived microbial metabolites in pancreatic ductal adenocarcinoma (PDAC) activated the aryl hydrocarbon receptor (AhR) in tumor-associated macrophages (TAMs) and suppressed CD8 T cell-mediated anti-tumor immunity (74). TAMs in TME can promote cancer progression through immune evasion. However, the involvement of tryptophan metabolism in the immunosuppressive function of TAMs in lung adenocarcinoma needs to be further investigated.

In recent years, various metabolism-related prediction models such as those based on glutamine metabolism (75), glycolysis (76), sphingolipid metabolism (77), lipid metabolism (53), and others have demonstrated the ability to predict the prognosis and immunotherapy of LUAD patients to some extent. Metabolic reprogramming plays an important role in tumorigenesis and development. In addition to using the Warburg effect generated by glycolysis to supply energy for themselves, tumor cells can also use metabolic reprogramming to obtain energy substances by pathways such as glutamine catabolism and fatty acid oxidation. In the study of XU et al., three subtypes were revealed by proteomics, which was associated with clinical and molecular features; among them, type I was closely related to cell metabolism and tumor microenvironment, which was mainly in the early clinical stage and had the most favorable prognosis (78). This represents a correlation between metabolic activation and good prognosis in lung adenocarcinoma, which coincides with the results of the GSVA-enriched correlation analysis of the five prognostic features we obtained. In addition, prognostic modeling has evolved rapidly in recent years, exploring the predictive power of cancer prognosis and immunotherapy at different levels, such as mining the potential predictive power of immunogenic death (ICD) (79), Treg cells (80), and purine metabolism (81) from single-cell sequencing data.

Taken together, our results suggest that TRP-associated prognostic modeling can be used to classify the prognosis of LUAD patients, contributing to a better understanding of the molecular mechanisms of LUAD and providing new insights into targeting and immunotherapy. As a hub gene associated with tryptophan metabolism, PTTG1 plays a promotive role in lung adenocarcinoma progression and is a potential predictive biomarker for clinical outcomes and immunotherapy response in lung adenocarcinoma, which requires further prospective studies and larger populations. Our study has potential shortcomings: first, “tryptophan metabolism” in this study mainly focuses on the expression level of tryptophan-related genes rather than tryptophan metabolizing enzyme activities or tryptophan fluxes, which is a limitation of the study of tryptophan metabolism in lung adenocarcinomas; second, it is a retrospective study, and multicenter cohort studies are needed to validate the predictive value of this TRP-associated prognostic model as a predictive biomarker of immunotherapeutic response in lung adenocarcinoma. In addition, further animal experiments are needed to explore the functional role of PTTG1 in lung adenocarcinoma, as well as to detect the specific effects of PTTG1 on tryptophan metabolism by methods such as liquid chromatography-tandem mass spectrometry (LC-MS/MS), which could help to provide stronger clues to guide the clinical application.

## Data availability statement

The datasets presented in this study can be found in online repositories. The names of the repository/repositories and accession number(s) can be found in the article/**Supplementary Material**.

## Ethics statement

The studies involving humans were approved by Institutional Review Board of Guilin Medical University Hospital (No. 2022YJSLL-78). The studies were conducted in accordance with the local legislation and institutional requirements. The participants provided their written informed consent to participate in this study.

## Author contributions

ZW: Conceptualization, Data curation, Formal analysis, Investigation, Methodology, Software, Visualization, Writing – original draft. JZ: Data curation, Methodology, Project administration, Supervision, Writing – original draft. CZ: Investigation, Methodology, Project administration, Software, Validation, Writing – original draft. HC: Data curation, Formal analysis, Investigation, Methodology, Writing – original draft. LW: Data curation, Methodology, Supervision, Writing – original draft. YX: Conceptualization, Formal analysis, Investigation, Methodology, Project administration, Validation, Writing – original draft. HM: Writing – review & editing, Methodology, Supervision, Validation, Visualization. SM: Funding acquisition, Resources, Software, Writing – review & editing, Data curation, Formal analysis, Investigation. XW: Funding acquisition, Project administration, Resources, Software, Writing – review & editing, Supervision. CL: Validation, Writing – review & editing, Funding acquisition, Investigation, Project administration, Resources, Software.
