# Supplementary material for: Identification and validation of tryptophan-related gene signatures to predict prognosis and immunotherapy response in lung adenocarcinoma reveals a critical role for PTTG1
Source: Front Immunol. 2024 Jul 31;15:1386427. doi: 10.3389/fimmu.2024.1386427 (PMC11321965; doi:10.3389/fimmu.2024.1386427)
Supplement: Supplementary file 1 [file DataSheet_1.docx]

**Supplementary Figure**


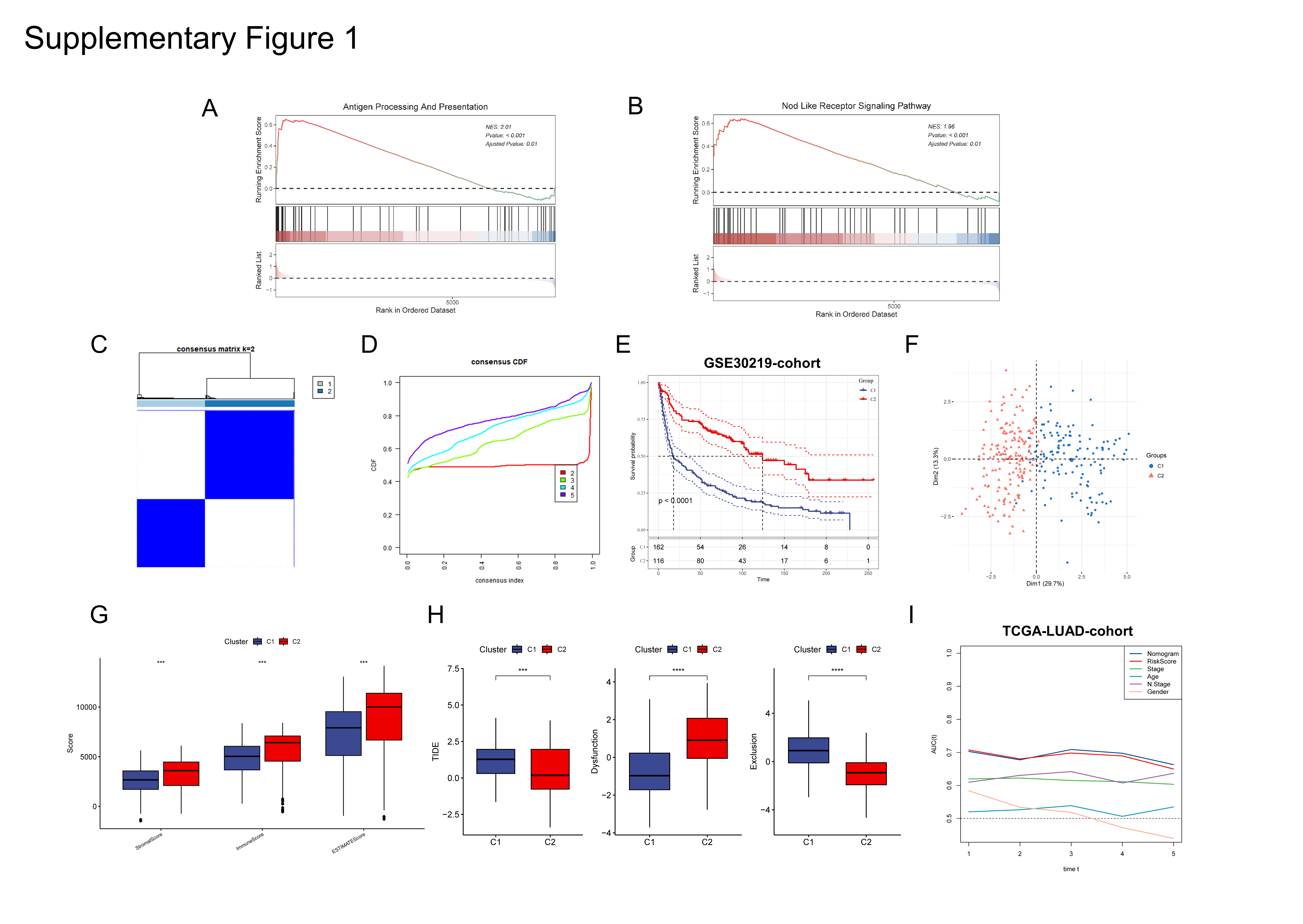


**Supplementary Figure 1** (A, B) Enrichment analysis of GSEA in the high TRP group. (C, D) Consensus heatmap matrix and consensus CDF curves for the GSE30219 cohort. (E) Survival analysis of the two clusters of the GSE30219 cohort (F) PCA analysis showing the distribution of the two clusters. (G) Stromal score, immune score, and estimate score of the two clusters. (H) TIDE scores of the two clusters. (I) Comparison of AUC values between column line graphs and other clinical features.





**Supplementary Figure 2** (A, D) Kaplan-Meier survival analysis of the TRP high- and low-risk groups. (B, E) Time-dependent ROC curve analysis in the GEO cohort. (C, F) Heatmap of risk score distribution, patient survival status and duration, and TRP metabolism-related gene expression in the GEO cohort. (G, H) Pathway enrichment analysis of high and low-risk scores. (I) Differences in abundance and immune function of tumor-infiltrating immune cells in high- and low-risk groups. (J) Correlation of risk scores with ICB response characteristics and each step of the tumor immune cycle.


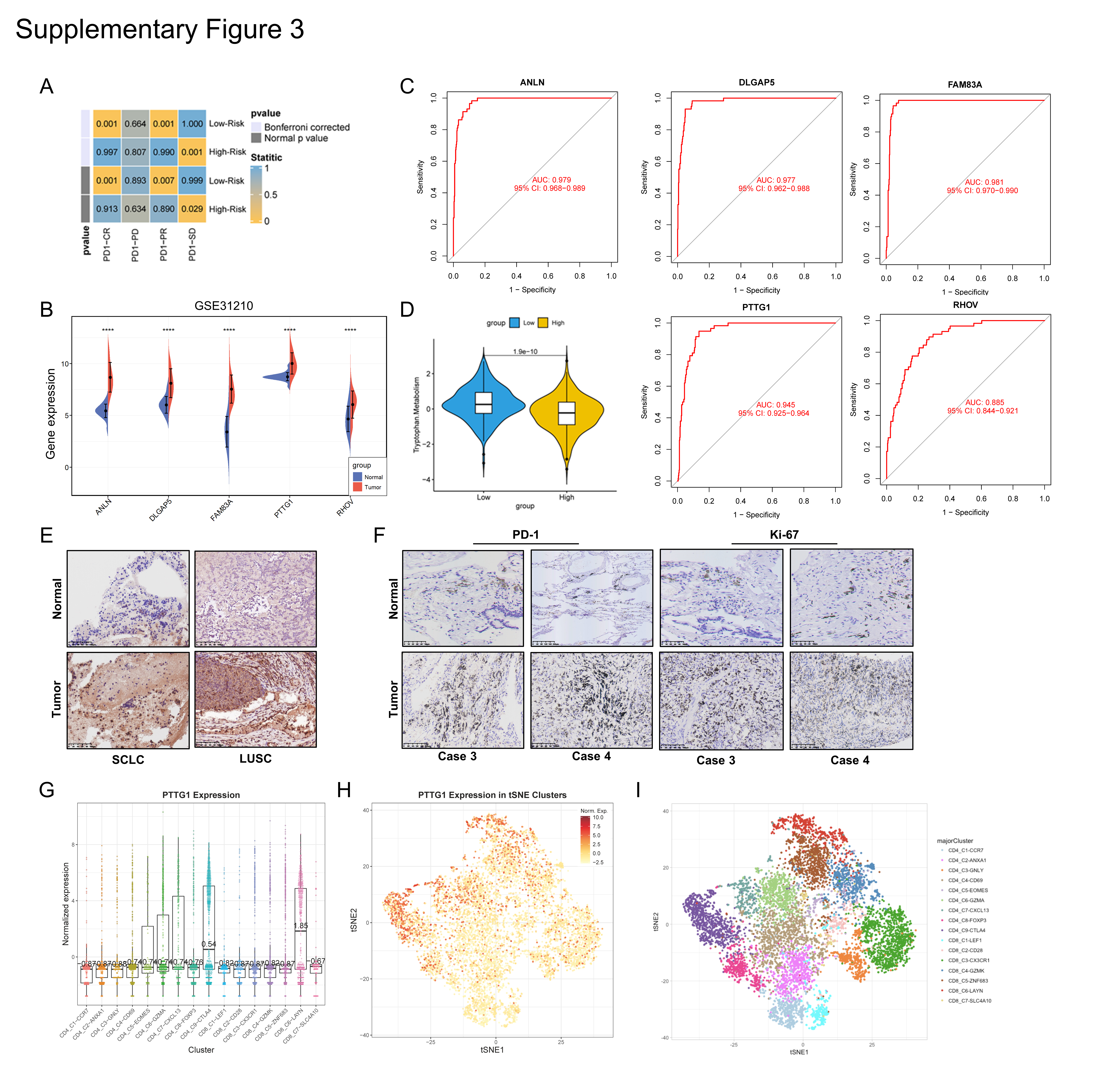


**Supplementary Figure 3** (A) Submap tool analysis showing that TRPRS can predict response to anti-PD-1 therapy. The obtained p-values were adjusted by the Bonferroni method. (B) Violin plot demonstrating the expression of five prognostic features in cancer and paracancer. (C) ROC diagnostic curves for the five prognostic features. (D) Difference in tryptophan metabolism score between high and low-risk groups (ssGSEA). (E, F) Representative images of IHC. (G-I) Differences in PTTG1 expression in T cell subsets.
